# Plasma Membrane Association but Not Midzone Recruitment of RhoGEF ECT2 Is Essential for Cytokinesis

**DOI:** 10.1016/j.celrep.2016.11.029

**Published:** 2016-12-06

**Authors:** Kristýna Kotýnková, Kuan-Chung Su, Stephen C. West, Mark Petronczki

**Affiliations:** 1 Cell Division and Aneuploidy Laboratory, Cancer Research UK London Research Institute, Clare Hall Laboratories, Blanche Lane, South Mimms, Herts EN6 3LD, UK; 2 DNA Recombination and Repair Laboratory, The Francis Crick Institute, 1 Midland Road, London NW1 1AT, UK; 3 Whitehead Institute and Department of Biology, MIT, 9 Cambridge Center, Cambridge, MA 02142, USA; 4 Boehringer Ingelheim RCV, Dr.-Boehringer-Gasse 5-11, 1121 Vienna, Austria

**Keywords:** cytokinesis, mitosis, cell division, plasma membrane, cytoskeleton, contractile ring, cleavage plane, spindle midzone, optogenetics, chemical genetics

## Abstract

Cytokinesis, the final step of cell division, begins with the formation of a cleavage furrow. How the mitotic spindle specifies the furrow at the equator in animal cells remains unknown. Current models propose that the concentration of the RhoGEF ECT2 at the spindle midzone and the equatorial plasma membrane directs furrow formation. Using chemical genetic and optogenetic tools, we demonstrate that the association of ECT2 with the plasma membrane during anaphase is required and sufficient for cytokinesis. Local membrane targeting of ECT2 leads to unilateral furrowing, highlighting the importance of local ECT2 activity. ECT2 mutations that prevent centralspindlin binding compromise concentration of ECT2 at the midzone and equatorial membrane but sustain cytokinesis. While the association of ECT2 with the plasma membrane is essential for cytokinesis, our data suggest that ECT2 recruitment to the spindle midzone is insufficient to account for equatorial furrowing and may act redundantly with yet-uncharacterized signals.

## Highlights

• Plasma membrane binding of RhoGEF ECT2 is essential for cytokinesis in human cells
• ECT2 membrane association at anaphase is required and sufficient for cell division
• Local optogenetic targeting of ECT2 to the membrane induces unilateral furrows
• BRCT mutations in ECT2 compromise midzone localization but support cytokinesis

## Graphical Abstract

**Figure:**
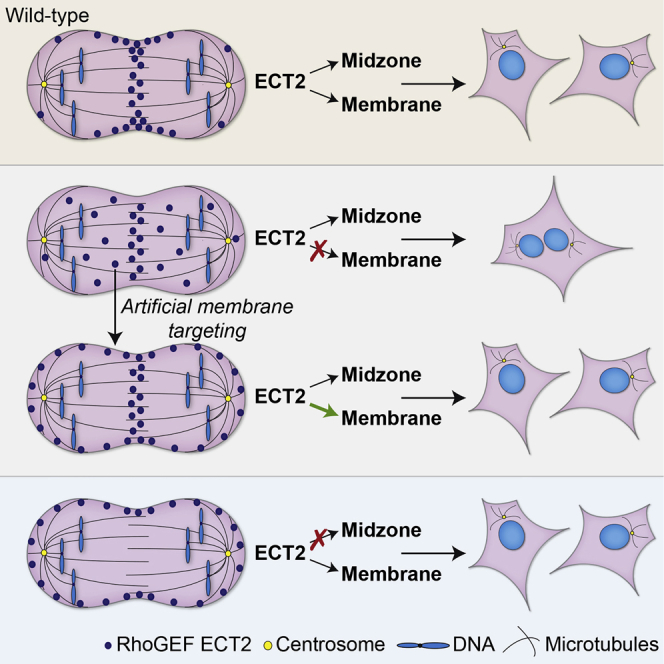

## Teaser

Kotýnková et al. investigate cell division in human cells. Using chemical genetics and optogenetics, they find that plasma membrane association of the RhoGEF ECT2 during anaphase is essential for cytokinesis. Contrary to current models, recruitment of ECT2 to the spindle midzone may not account for equatorial cleavage plane specification.

## Introduction

Cytokinesis is the final step of cell division that partitions sister genomes into the two nascent daughter cells (Morgan, 2006). Temporal and spatial regulation of cytokinesis prevents chromosomal instability and the unequal partitioning of organelles (Fujiwara et al., 2005, Ganem et al., 2007, Ganem et al., 2009, Lacroix and Maddox, 2012). Temporal regulation is ensured by Cdk1, which inhibits cytokinesis before anaphase onset. Spatially, the cleavage plane needs to be positioned at the cell equator to accurately partition sister genomes and to yield equally sized progeny (D’Avino et al., 2015, Fededa and Gerlich, 2012, Morgan, 2006).

In animal cells, the cleavage plane is specified during anaphase by the mitotic spindle apparatus (Burgess and Chang, 2005, Rappaport, 1996). Two spindle substructures are thought to position the cytokinetic furrow at the equatorial cortex: astral microtubules and the spindle midzone, a structure formed by antiparallel microtubules between segregating sister genomes in anaphase (Bringmann and Hyman, 2005, Dechant and Glotzer, 2003). The underlying molecular mechanisms are not well understood. Strong evidence suggests that signals from the mitotic spindle apparatus lead to the local activation of the small guanosine triphosphatase (GTPase) RhoA (Jordan and Canman, 2012, Piekny et al., 2005). The active guanosine triphosphate (GTP)-bound form of RhoA promotes contractile ring formation and furrow ingression by simultaneous activation of actin assembly and non-muscle myosin II activity (Bement et al., 2005, Piekny et al., 2005, Yoshizaki et al., 2003). Experimentally induced local RhoA activation at the plasma membrane can induce furrowing in a spindle- and cell-cycle-independent manner (Wagner and Glotzer, 2016), highlighting the need to understand the control of RhoA activity during cytokinesis. How the mitotic spindle breaks cortical isotropy to generate an equatorial contractile zone and thereby positions the cleavage plane is one of the major unresolved questions in cell biology.

The guanine nucleotide exchange factor (GEF) activating RhoA during cytokinesis is a conserved protein called ECT2. Mutation or depletion of ECT2 prevents contractile ring assembly and cleavage furrow formation, resulting in cytokinesis failure (Dechant and Glotzer, 2003, Prokopenko et al., 1999, Tatsumoto et al., 2003, Yüce et al., 2005).

**Figure 1:**
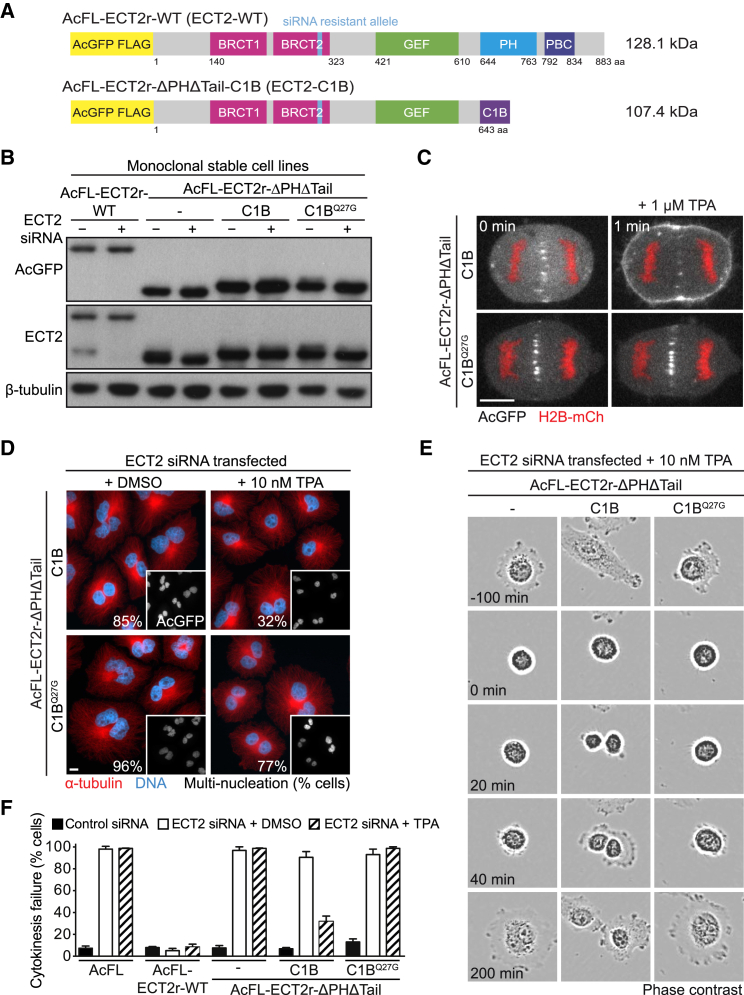
Membrane Association of ECT2 Is Essential for Cytokinesis (A) Domain organization of AcFL-tagged and siRNA-resistant full-length ECT2-WT protein (reference sequence NCBI: NP_001245245) and ECT2-C1B protein. (B) Immunoblot analysis of protein lysates from cells stably expressing the indicated transgenes. Lysates were prepared 48 hr after transfection with NTC (−) or ECT2 siRNA (+). The transgene is expressed in more than 95% of cells. (C) Live-cell imaging of ECT2-C1B proteins. Stable cell lines expressing ECT2-C1B or ECT2-C1B^Q27G^ (white) were transiently transfected with H2B-mCherry (red). Cells were imaged 48 hr after transfection and treated with TPA at t = 0 min. Scale bars in this and the following panels represent 10 μm. (D) Immunofluorescence (IF) analysis of ECT2-C1B cell lines. TPA or DMSO was added 6 hr after transfection with ECT2 siRNA. Multi-nucleation was analyzed 48 hr after transfection (n > 300 cells each from three independent experiments). (E) Live-cell imaging of indicated cell lines. Cells were transfected with ECT2 siRNA and treated with TPA as in (D). Cells were imaged from 24 hr after transfection. Time point t = 0 min was set to the metaphase-to-anaphase transition. (F) Quantification of cytokinetic phenotypes using live-cell imaging as described in (E). Mono-nucleated cells undergoing cell division were scored from 24 to 72 hr after transfection (n > 100 each, bars represent mean ± SD of three independent experiments). See also Figure S1.

The essential cytokinetic function and properties of ECT2 (Figure 1A) put it in a central position in models of how the spindle midzone promotes the position of the cytokinetic furrow. First, ECT2 contains a central GEF domain that is responsible for RhoA activation and required for cytokinesis (Prokopenko et al., 1999, Rossman et al., 2005, Su et al., 2011). Second, ECT2 localizes to the spindle midzone upon anaphase onset via the interaction of its N-terminal BRCA1 C-terminal (BRCT) domains (Leung and Glover, 2011) with MgcRacGAP (also called CYK-4 or RACGAP1), a subunit of the centralspindlin complex (Mishima et al., 2002, Pavicic-Kaltenbrunner et al., 2007, Somers and Saint, 2003, Yüce et al., 2005, Zhao and Fang, 2005). This interaction requires MgcRacGAP phosphorylation by the mitotic kinase PLK1 (Burkard et al., 2009, Wolfe et al., 2009, Yüce et al., 2005). Inhibition of PLK1, as well as replacement of MgcRacGAP with a non-phosphorylatable mutant, abrogates ECT2 accumulation at the spindle midzone, RhoA activation, and furrow ingression (Burkard et al., 2009, Petronczki et al., 2007, Wolfe et al., 2009). Thus, complex formation between ECT2 and MgcRacGAP and consecutive recruitment of the RhoGEF to the midzone are thought to be crucial for cytokinesis. Finally, ECT2 interacts with the plasma membrane upon anaphase onset (Chalamalasetty et al., 2006, Su et al., 2011). This association is directed by two C-terminal regions of ECT2, a pleckstrin homology (PH) domain and a cluster of basic amino acids (PBC) (Figure 1A). Deletion of these regions abrogates ECT2’s membrane recruitment, RhoA activation, and cleavage furrow formation (Su et al., 2011), indicating that the association of the RhoGEF protein with the cell periphery might be essential for cytokinesis. ECT2 is distributed throughout the cytoplasm in metaphase and associates with the plasma membrane only after anaphase onset and CDK1 inactivation. Although initially distributed evenly across the cell periphery, ECT2 accumulates at the equatorial region of the membrane at the time of furrow ingression (Su et al., 2011). The equatorial enrichment of ECT2 was shown to require the centralspindlin complex and PLK1 enzymatic function, both of which are essential for the spindle midzone recruitment of the GEF protein (Yüce et al., 2005, Petronczki et al., 2007, Burkard et al., 2009).

Models proposed that accumulation of ECT2 to the spindle midzone and equatorial plasma membrane could lead to the preferential activation of RhoA and positioning of the cleavage furrow at the equator (D’Avino et al., 2015, Fededa and Gerlich, 2012, Su et al., 2011). Predictions of this model are that GEF activity, midzone association, plasma membrane binding, and equatorial enrichment of ECT2 are essential prerequisites for cytokinesis. Existing data strongly support the requirement for ECT2’s GEF activity during cytokinesis (Prokopenko et al., 1999, Su et al., 2011). We set out to interrogate ECT2’s association with the spindle midzone and the plasma membrane.

## Results

### Plasma Membrane Association of ECT2 Is Essential for Cytokinesis in Human Cells

Previous work employed an ECT2 variant lacking the C-terminal 252 amino acids (ECT2-ΔPHΔTail) to suggest that the translocation of ECT2 to the plasma membrane is a prerequisite for cytokinesis (Su et al., 2011). The large deletion could compromise other functions of the molecule and thereby contribute to the observed phenotype. To determine whether the association of ECT2 with the membrane is a requirement for cleavage furrow formation in human cells, we employed a chemical genetic system that allowed us to artificially control the association of ECT2 with the plasma membrane. The system is based on hybrid proteins containing a typical C1 domain from human protein kinase Cα (PKCα) (Colón-González and Kazanietz, 2006) that binds the plasma membrane via interaction with diacylglycerol or phorbol esters (Colón-González and Kazanietz, 2006, Lekomtsev et al., 2012).

We generated a monoclonal stable HeLa Kyoto cell line expressing chimeric ECT2-C1B, in which the entire C-terminal part of ECT2, including the PH domain and PBC, was replaced by the C1B domain from human PKCα (Figures 1A and 1B; Figure S1A). The ECT2-C1B transgene was tagged with AcGFP (*Aequorea coerulescens* GFP)-FLAG (AcFL) and was rendered resistant to ECT2 small interfering RNA (siRNA) by inclusion of synonymous nucleotide changes. The hybrid ECT2-C1B protein rapidly associated with the plasma membrane in anaphase cells after addition of the phorbol ester 12-*O*-tetradecanoylphorbol-13-acetate (TPA) to the cell medium (Figure 1C; Movie S1). Despite membrane translocation, the hybrid protein remained detectable at the spindle midzone in anaphase cells. Mutation of C1B glutamine 27 to glycine (Q27G) (Figure S1B), a change predicted to disrupt the interaction with phorbolesters (Bögi et al., 1999, Colón-González and Kazanietz, 2006), abrogated the translocation of the hybrid ECT2-C1B protein to the plasma membrane (Figure 1C; Figures S1C–S1E; Movies S1 and S2).

To determine whether artificial membrane recruitment of ECT2 supports cytokinesis in the absence of the protein’s normally essential native membrane engagement domains, ECT2-C1B-expressing cells were transfected with ECT2 siRNA to deplete endogenous protein and treated with 10 nM TPA. Multi-nucleation was determined as readout for cytokinesis failure. In the presence of the solvent, DMSO, most ECT2-C1B-expressing cells were converted into multi-nucleated cells upon depletion of the endogenous protein (Figure 1D). Strikingly, addition of TPA strongly suppressed the fraction of multi-nucleated cells (Figure 1D). TPA treatment had only a minor effect in cells expressing ECT2-C1B^Q27G^, indicating that the rescue effect is dependent on TPA-induced membrane association of the C1B domain. To assess the execution of cytokinesis directly, we used live-cell imaging. Expression of a wild-type (WT) ECT2 transgene but not an ECT2 version lacking the PH domain and PBC supported cell division following depletion of the endogenous protein (Figures 1E and 1F). Addition of DMSO or TPA had no significant effect on cytokinesis in these situations. TPA addition, but not DMSO addition, allowed most ECT2-C1B-expressing cells to successfully divide, while >98% of the ECT2-C1B^Q27G^-expressing cells failed cytokinesis despite TPA addition (Figures 1E and 1F). We conclude that the interaction of the RhoGEF ECT2 with the plasma membrane is a key property of ECT2 that is indispensable for the execution of cytokinesis in somatic human cells.

### Plasma Membrane Association of ECT2 from Anaphase Onward Is Required and Sufficient for Cytokinesis

**Figure 2:**
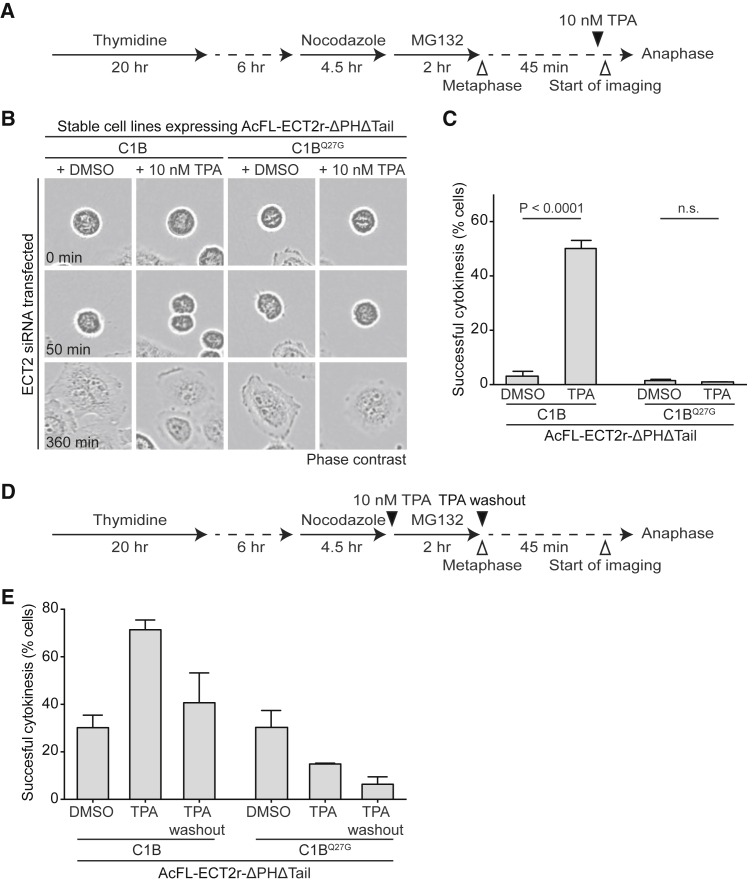
Plasma Membrane Association of ECT2 in Anaphase Is Required and Sufficient to Support Cytokinesis (A) Synchronization scheme for anaphase-specific membrane targeting of ECT2-C1B proteins. (B) Live-cell imaging of ECT2-C1B and ECT2-C1B^Q27G^ cell lines. Cells were transfected with ECT2 siRNA and synchronized in metaphase using the protocol depicted in (A). Cells were treated with DMSO or TPA 45 min after release from metaphase and imaged using bright-field microscopy. Time point t = 0 min was set to the metaphase-to-anaphase transition. (C) Quantification of cytokinetic phenotypes of cells recorded in (B). Mono-nucleated cells that were in metaphase at the beginning of recording were scored (n > 200 each, bars represent mean ± SD of three independent experiments, Student’s t test). (D) Synchronization scheme for membrane targeting of ECT2-C1B proteins in metaphase followed by TPA washout before anaphase onset. (E) Quantification of cytokinetic phenotypes of cells treated as shown in (D). Mono-nucleated cells that were in metaphase at the beginning of recording were scored (n > 65 each, bars represent mean ± SD of three independent experiments). See also Figure S2.

The C1B hybrid system can be employed to temporally dissect the requirement for ECT2’s association with the cell envelope. To target ECT2 to the plasma membrane at the metaphase-to-anaphase transition, we combined depletion of endogenous ECT2 with cell synchronization (Figure 2A). Following the release of ECT2-C1B-expressing cells from metaphase, DMSO or 10 nM TPA were added to the cell medium and cells were tracked through cell division by live-cell imaging. While almost all DMSO-treated cells expressing ECT2-C1B failed to undergo cytokinesis, addition of TPA restored cell division in about half of the cell population (Figures 2B and 2C). This rescue effect was abolished in cells expressing the ECT2-C1B^Q27G^ hybrid protein. The TPA-dependent rescue effect in ECT2-C1B-expressing cells was also observed with 250 nM TPA, a concentration at which we can clearly detect plasma membrane translocation of the hybrid protein (Figures S2A and S2B). These data show that the association of the RhoGEF ECT2 with the plasma membrane at anaphase onset can support cleavage furrow formation and cytokinesis.

Next, we tested whether ECT2’s engagement with the plasma membrane only before anaphase onset supports cytokinesis. ECT2-C1B-expressing cells were depleted of endogenous ECT2, synchronized, and treated with DMSO or TPA from prometaphase to metaphase (Figure 2D). At release into anaphase, TPA was either removed or maintained (Figure 2D). Washout of TPA reduced the fraction of ECT2-C1B-expressing cells that underwent successful cytokinesis close to the fraction observed in DMSO-treated control cells (Figure 2E). We conclude that the association of ECT2 with the plasma membrane from the metaphase-to-anaphase transition onward is not only sufficient but also required for the execution of cell division. These experiments define the essential time window for RhoA activation by the RhoGEF ECT2 at the plasma membrane during cytokinesis.

### Membrane Targeting of ECT2’s GEF Domain Alone Cannot Support Cytokinesis

**Figure 3:**
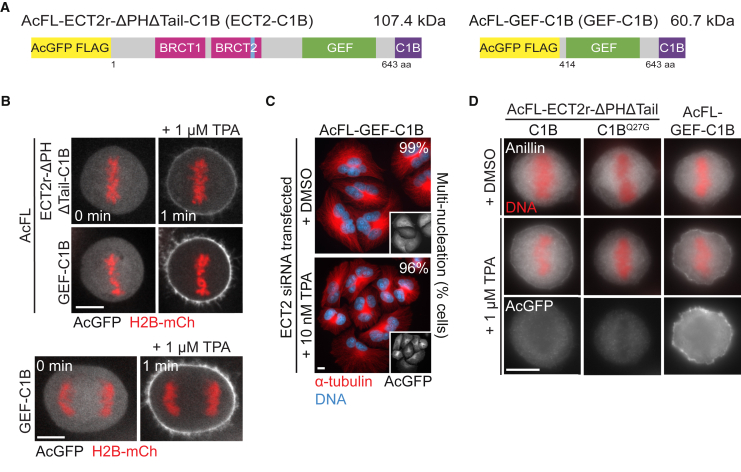
Membrane Targeting of ECT2’s GEF Domain Does Not Support Cytokinesis (A) Domain organization of transgenic C1B proteins. (B) Live-cell imaging of ECT2-C1B and GEF-C1B proteins in metaphase (upper panel) and GEF-C1B in anaphase (lower panel). Cells stably expressing the indicated transgenes were transfected with H2B-mCherry (red) and imaged 48 hr after transfection. Cells were treated with TPA at t = 0 min. The scale bars in this and the following panels represent 10 μm. (C) IF analysis of cells stably expressing GEF-C1B. DMSO or TPA was added to the cells 6 hr after transfection with ECT2 siRNA. Multi-nucleation was analyzed 48 hr after transfection (n > 300 cells each from three independent experiments). (D) IF analysis of anillin in metaphase cells expressing the indicated transgenes. Cells were treated with nocodazole to enrich the population for prometaphase cells. One hour after nocodazole washout, the cells were treated with DMSO or TPA for 5 min and analyzed. Membrane localization of ECT2 variants is largely lost upon cell fixation. See also Figure S3.

We next set out to determine whether the RhoGEF activity and the ability to associate with the plasma membrane are the only two key functions of ECT2 during cytokinesis. For these experiments, a stable cell line expressing the GEF domain of ECT2 fused to the C1B domain (GEF-C1B) was generated (Figure 3A; Figure S3A). The GEF-C1B hybrid protein rapidly and efficiently translocated to the plasma membrane in metaphase and anaphase cells upon treatment with TPA (Figure 3B). In contrast to the ECT2-C1B protein (Figure 1), artificial membrane targeting of the GEF domain alone was unable to complement the loss of endogenous ECT2 and to accumulate at the spindle midzone (Figures 3B and 3C). Thus, while the GEF activity of ECT2 is essential for cell division (Prokopenko et al., 1999, Somers and Saint, 2003, Su et al., 2011, Tatsumoto et al., 2003, Yüce et al., 2005), the artificial targeting of the GEF domain alone to the plasma membrane is insufficient to support cell division. Consistent with other studies (Kim et al., 2005, Wagner and Glotzer, 2016), these results suggest that the N-terminal region of ECT2 that contains the BRCT repeats and is absent in the GEF-C1B protein might play an important role during cytokinesis.

### Forced Membrane Recruitment of ECT2 and Its GEF Domain in Metaphase Elicits Signs of Contractility

During cytokinesis, ECT2 translocates to the plasma membrane at the time of anaphase onset when Cdk1 activity declines (Su et al., 2011). Cdk1 activity has emerged as a potent inhibitor of cleavage furrow formation (Niiya et al., 2005, Potapova et al., 2006). Using the C1B hybrid system to override the control mechanism exerted by Cdk1, we therefore tested whether ECT2’s association with the plasma membrane restricts cleavage furrow formation to anaphase. We forced ECT2-C1B and GEF-C1B fusion proteins to the plasma membrane prematurely in metaphase cells and scored for signs of contractility by analyzing RhoA and the contractile ring protein anillin (Figure 3D; Figure S3B) (Bement et al., 2005, Liu et al., 2012, Piekny et al., 2005, Piekny and Glotzer, 2008). We quantified the ratio of fluorescence intensity at the cell periphery and in the cytoplasm for both proteins (Figures S3C and S3D). We observed a small but significant increase of anillin and RhoA membrane signals in metaphase cells after ECT2-C1B and GEF-C1B membrane targeting (Figure 3D; Figures S3B–S3D). GEF-C1B-expressing cells also exhibited signs of hypercontractility, as judged by the irregular shape of the cell boundary (Figure 3D), a phenotype that was not observed after ECT2-C1B membrane targeting. Consistent with RhoA activation experiments (Wagner and Glotzer, 2016), this suggests that preventing ECT2 membrane translocation in metaphase contributes to restricting contractility and furrowing activity to anaphase. Our data also provide strong support for a role of the BRCT domains in intramolecularly restricting RhoGEF activity of ECT2 (Kim et al., 2005).

### Optogenetic Targeting of ECT2 to the Plasma Membrane Induces Cleavage Furrow Formation

To interrogate the importance of the spatial distribution of ECT2 at the cell membrane, we expanded our analysis by employing an optogenetic system based on the photosensitive cryptochrome protein (Cry2) from *Arabidopsis thaliana*. Upon blue-light illumination, Cry2 changes its conformation and interacts with the CIB1 protein, establishing an optically controlled dimerization system (Kennedy et al., 2010).

**Figure 4:**
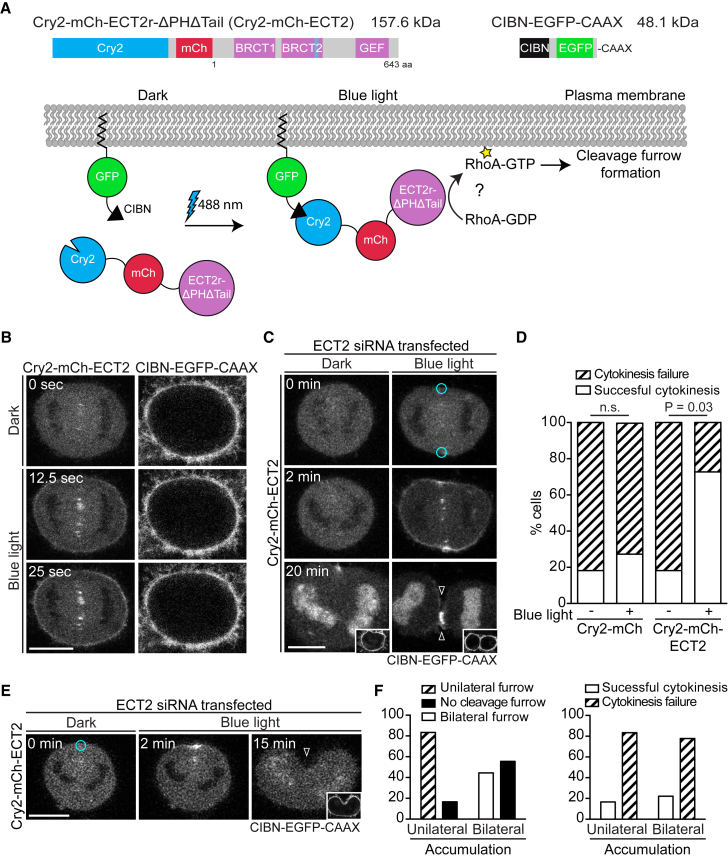
Optogenetic Targeting of ECT2 to the Plasma Membrane Induces Cleavage Furrow Formation (A) Schematic depiction of optogenetic targeting of Cry2-mCh-ECT2 to the plasma membrane. (B) Frames from confocal live-cell imaging. Cells were transfected with Cry2-mCh-ECT2 and CIBN-EGFP-CAAX. Cells were imaged 48 hr post-transfection, and the whole field was activated by scanning with a 488 nm laser at t = 0 s. Scale bars in this and the following panels represent 10 μm. (C) Live-cell imaging with or without blue-light illumination. Cells stably expressing CIBN-EGFP-CAAX (inset) were transfected with Cry2-mCh-ECT2 and ECT2 siRNA and imaged 24 hr after siRNA transfection. Photoactivation was performed by illumination with a 488 nm laser within two small circular regions at the equatorial periphery, as marked in the image. Cleavage furrow ingression is indicated by open arrowheads. (D) Quantification of cytokinetic phenotypes after optogenetic membrane targeting of ECT2 as described in (C). Metaphase or early anaphase cells with or without blue-light illumination were scored (n = 11, Fisher’s exact test). (E) Live-cell imaging with unilateral blue-light illumination. Cells stably expressing CIBN-EGFP-CAAX (inset) were transfected with Cry2-mCh-ECT2 and ECT2 siRNA. Cells were imaged 24 hr after siRNA transfection. Photoactivation was induced by unilateral illumination with a 488 nm laser within the circular region at the equatorial periphery as indicated. (F) Quantification of the furrow formation phenotype (left) and cytokinetic phenotype (right) in relation to protein distribution after unilateral membrane targeting of ECT2 as described in (E). Anaphase cells were scored (n = 15). See also Figure S4.

For optogenetic targeting experiments, a cell line stably expressing the N-terminal part of CIB1 protein fused to EGFP (CIBN-EGFP) was generated. A C-terminal CAAX prenylation motif was added to anchor the protein at the plasma membrane (CIBN-EGFP-CAAX) (Figure 4A). We then fused a truncated version of ECT2 lacking the protein’s native membrane engagement domains to Cry2-mCherry to create a photoresponsive Cry2-mCh-ECT2r-ΔPHΔTail fusion protein (Cry2-mCh-ECT2) (Figure 4A). To test the system, we transfected CIBN-expressing cells with Cry2-mCh-ECT2 and imaged them using confocal microscopy. Upon whole-cell illumination with a 488 nm laser, the Cry2-mCh-ECT2 protein rapidly translocated to the plasma membrane and co-localized with CIBN in anaphase cells (Figure 4B). Subsequently, we determined whether optogenetic targeting of the Cry2-mCh-ECT2 protein to the plasma membrane supports cleavage furrow formation and cytokinesis after depletion of endogenous ECT2 protein. We induced the interaction of Cry2-mCh-ECT2 with plasma membrane-bound CIBN in metaphase or early anaphase cells by repeated illumination with a 488 nm laser in circular regions at two opposite locations of the equatorial cortex. Upon illumination, we observed that the Cry2-mCh-ECT2 protein was partially depleted from the cytoplasm and rapidly translocated to the plasma membrane, concentrating mainly at the equatorial periphery (Figure 4C; Movie S3). In contrast, we did not detect any plasma membrane recruitment of the fusion protein without blue-light illumination. Strikingly, optogenetic targeting of Cry2-mCh-ECT2 to the plasma membrane restored cleavage furrow ingression and cytokinesis in more than 70% of cells, despite the absence of essential membrane engagement domains of ECT2 (Figures 4C and 4D; Movie S3). Rescue activity was dependent on illumination with blue light. Membrane targeting of the control protein Cry2-mCh was unable to rescue cytokinesis after depletion of endogenous ECT2 with or without the blue-light activation (Figure 4D). In support of our chemical genetic experiments, the optogenetic targeting approach demonstrates that association of ECT2 with the plasma membrane in anaphase is a key step for cytokinesis.

Blue-light illumination of a region close to a cell pole did not result in the local polar accumulation of Cry2-mCh-ECT2 or polar furrowing events (8/8 recorded cells) (Figure S4). In most cells with polar illumination (5/8), the fusion protein accumulated at the equator; this was accompanied by equatorial furrow formation (Figure S4). We next determined whether Cry2-mCh-ECT2 targeting to only one area of the cleavage plane causes unilateral furrowing. Anaphase cells depleted of endogenous ECT2 were illuminated in one region of the equatorial periphery (Figure 4E; Movie S4). In 60% of the cells, diffusion of Cry2-mCh-ECT2 along the cell membrane or isotropic binding to the membrane prevented unilateral accumulation of the fusion protein. This ECT2 localization, while reminiscent of the bilateral targeting, did not allow successful completion of cytokinesis despite bilateral furrowing in a fraction of the cells (Figure 4F). In the remaining 40% of cells, localized accumulation of Cry2-mCh-ECT2 was observed at the site of unilateral illumination. Strikingly, the unilateral equatorial accumulation of Cry2-mCh-ECT2 was associated with the formation of a unilateral furrow at the site of enrichment of the fusion protein (Figures 4E and 4F; Movie S4). This experiment suggests that the local activity of ECT2 at the plasma membrane is necessary to drive cleavage furrow formation.

### Mutations in the BRCT1 Domain Prevent the Interaction of ECT2 with MgcRacGAP

**Figure 5:**
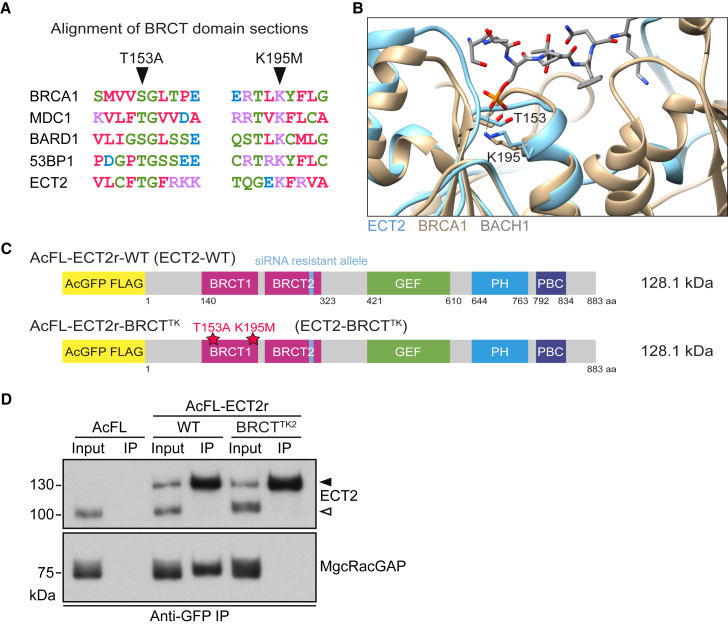
Mutations in the BRCT1 Domain of ECT2 Disrupt Binding to MgcRacGAP (A) Sequence alignment of indicated human BRCT-domain containing proteins. Conserved residues T153 and K195 are highlighted. (B) The crystal structure of the BRCT domains of ECT2 (light blue) (PDB: 4N40) (Zou et al., 2014) was aligned with a structure of the BRCT domains of BRCA1 (gold) bound to a BACH1 phosphopeptide (gray) (PDB: 1T15) (Clapperton et al., 2004). BRCA1 residues interacting with the phosphoserine of BACH1 and their ECT2 counterparts (T153 and K195) are highlighted. Hydrogen bonds are shown as dashed lines. (C) Domain organization of ECT2-WT protein and ECT2-BRCT^TK^ protein. (D) Protein extracts prepared from cells synchronized in anaphase that stably express the indicated proteins were subjected to anti-GFP immunoprecipitation (IP). Lysates (input) and IP fractions (20× input) were analyzed by immunoblotting.

**Figure 6:**
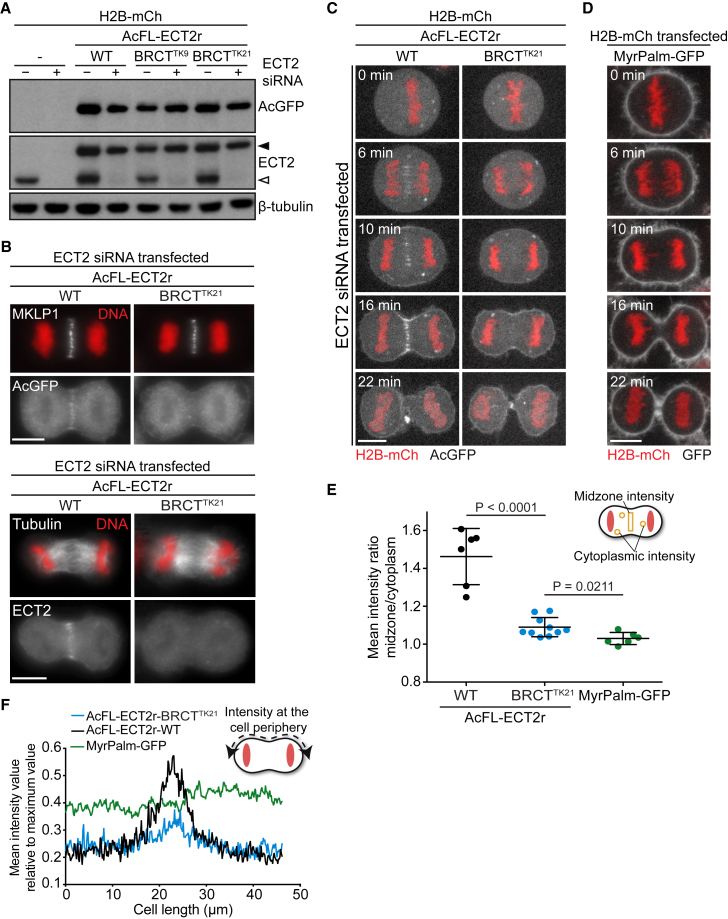
BRCT1 Mutations Compromise Spindle Midzone Localization and Equatorial Enrichment at the Plasma Membrane of ECT2 (A) Immunoblot analysis of protein lysates from the cell lines stably expressing the indicated transgenes. Lysates were prepared 48 hr after transfection with NTC (−) or ECT2 siRNA (+). Endogenous ECT2 protein and transgenic AcFL-ECT2r are indicated by open and filled arrowheads, respectively. All stable cell lines express the transgenes in more than 95% of cells. (B) IF analysis of spindle midzone localization of ECT2 in anaphase cells stably expressing ECT2-WT or ECT2-BRCT^TK^. Cells were transfected with ECT2 siRNA and synchronized using a thymidine release protocol. Cells were fixed and stained 36 hr after transfection. The scale bars in this and the following panels represent 10 μm. (C) Live-cell imaging of stable cell lines co-expressing the indicated transgenes. Cells were transfected with ECT2 siRNA and imaged 48 hr after transfection. Time point t = 0 min was set to the metaphase-to-anaphase transition. (D) Live-cell imaging of cells stably expressing MyrPalm-GFP. Cells were imaged 48 hr after transfection with H2B-mCherry. Time point t = 0 min was set to the metaphase-to-anaphase transition. (E) Quantification of the midzone localization of ECT2-WT, ECT2-BRCT^TK^, and MyrPalm-GFP in anaphase based on live-cell imaging data. The fluorescent intensity ratio between the midzone and the cytoplasm is plotted for individual cells (lines represent mean ± SD). (F) Quantification of the fluorescent intensity profile along the cell membrane of ECT2-WT, ECT2-BRCT^TK^, and MyrPalm-GFP proteins in anaphase cells. Values are plotted as the mean intensity value against the measured length (n = 6 cells for ECT2-WT and MyrPalm-GFP and n = 10 cells for ECT2-BRCT^TK^). See also Figure S5.

**Figure 7:**
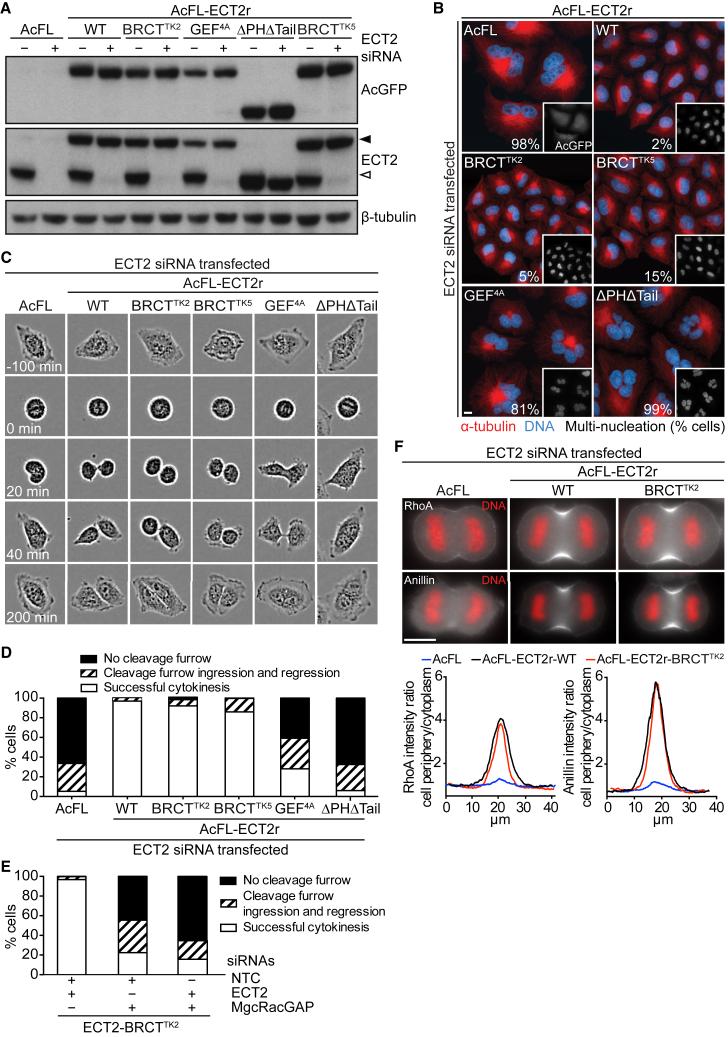
ECT2-BRCT^TK^ Supports Cytokinesis (A) Immunoblot analysis of protein lysates from monoclonal cell lines stably expressing the indicated transgenes. Lysates were prepared 48 hr after transfection with NTC (−) or ECT2 siRNA (+). Endogenous ECT2 protein and transgenic AcFL-ECT2r are indicated by open and filled arrowheads, respectively. All stable cell lines express the transgenes in more than 95% of cells. (B) IF analysis of indicated cell lines. Cells were transfected with ECT2 siRNA. Multi-nucleation levels were analyzed 48 hr after transfection (n > 300 cells each from three independent experiments). The scale bars in this panel and the following panels represent 10 μm. (C) Representative images showing cytokinetic phenotypes for indicated cell lines after depletion of endogenous ECT2. Cells were imaged with bright field microscopy from 24 hr after siRNA transfection onward. Time point t = 0 min was set to the metaphase-to-anaphase transition. (D) Quantification of cytokinetic phenotype from recordings in (C). Mono-nucleated cells undergoing cell division were scored from 24 to 48 hr post-transfection (n > 300 each, bars represent mean values of three independent experiments). (E) Quantification of cytokinetic phenotype of cells stably expressing ECT2-BRCT^TK^ and transfected with different siRNA combinations as indicated in the graph. Cells were imaged with bright field microscopy from 24 hr after transfection onward. Mono-nucleated cells undergoing cell division were scored from 24 to 60 hr post-transfection (n > 100 cells each, bars represent mean values of three independent experiments). (F) IF analysis and quantification of RhoA and anillin localization in anaphase cells expressing the AcFL tag, ECT2-WT, or ECT2-BRCT^TK^. Cells were transfected with ECT2 siRNA and synchronized by thymidine release protocol. Cells were fixed and stained 36 hr after transfection. The fluorescent intensity profile along the cell membrane for RhoA and anillin in anaphase cells is plotted as the ratio of the mean signal intensity at the cell periphery and the cytoplasm against the measured length (n = 15 cells, lines represent mean values). See also Figure S6.

Although the BRCT domain-mediated and PLK1-dependent interaction of ECT2 with its spindle midzone anchor MgcRacGAP is central to models of cleavage furrow positioning and ingression (Burkard et al., 2009, Petronczki et al., 2007, Su et al., 2011, Wolfe et al., 2009, Yüce et al., 2005), the importance of this interaction during cytokinesis has not been tested by altering full-length ECT2. Therefore, we sought to prevent this interaction by mutating residues T153 and K195 in the first BRCT domain of ECT2, which are conserved throughout different BRCT-containing proteins (Figure 5A). Structural analyses of other BRCT proteins indicate the importance of these residues in coordination of the phosphate in the BRCT-interacting peptide (Clapperton et al., 2004, Leung and Glover, 2011). An overlay of the X-ray structure of ECT2’s BRCT repeats (Zou et al., 2014) with the structure of BRCA1 bound to a BACH1 phosphopeptide (Clapperton et al., 2004) indicates that residues T153 and K195 in ECT2 are positioned suitably for phosphate coordination (Figure 5B). Mutation of T153 and K195 disrupts the localization of a transiently expressed N-terminal fragment of ECT2 to the spindle midzone and abrogates the interaction of recombinant ECT2 BRCT domains with cellular MgcRacGAP in pull-down experiments and with an MgcRacGAP phosphopeptide in biophysical assays (Wolfe et al., 2009, Zou et al., 2014). To investigate the role of the BRCT1 domain-mediated interaction of ECT2 with centralspindlin in cells, we introduced T153A and K195M mutations into a full-length siRNA-resistant and AcGFP-FLAG-tagged ECT2 construct (AcFL-ECT2r-BRCT^TK^ encoding ECT2-BRCT^TK^) (Figure 5C). We generated cell lines stably expressing the ECT2-BRCT^TK^ protein at a level close to the endogenous protein (Figures 6A and 7A). Immunoprecipitation (IP) of AcFL-tagged transgenic ECT2 proteins from protein extracts prepared from cells synchronized in anaphase revealed that ECT2-WT, but not ECT2-BRCT^TK^, was associated with MgcRacGAP (Figure 5D). This experiment demonstrates that the BRCT1 mutations T153A and K195M strongly inhibit the interaction of full-length ECT2 with the centralspindlin subunit MgcRacGAP in cell extracts.

### Mutations in the BRCT1 Domain Disrupt ECT2’s Spindle Midzone Localization and Enrichment at the Equatorial Plasma Membrane

To investigate the functional role of the BRCT1 domain-mediated interaction of ECT2 with centralspindlin during cytokinesis, we analyzed ECT2-WT- and ECT2-BRCT^TK^-expressing cell lines after depletion of endogenous ECT2 protein (Figures 5C and 6A).

To test whether the BRCT mutations T153A and K195M abolish ECT2 localization to the spindle midzone, anaphase cells were stained for transgenic or total ECT2 after depletion of the endogenous counterpart. While transgenic ECT2-WT co-localized with MKLP1, a subunit of the centralspindlin complex (colocalization in 8/8 cells) (White and Glotzer, 2012), ECT2-BRCT^TK^ was not enriched at the spindle midzone (colocalization in 3/25 cells) (Figure 6B, upper panel). Co-staining of total ECT2 with tubulin confirmed that ECT2-WT (24/24 cells), but not ECT2-BRCT^TK^ (4/36 cells), was recruited to the spindle midzone (Figure 6B, lower panel). Thus, BRCT1 domain mutations that prevent binding of ECT2 to MgcRacGAP abrogate ECT2’s localization to the spindle midzone in fixed cells.

Subsequently, ECT2 localization was tracked through cell division in live cells. Both ECT2-WT and ECT2-BRCT^TK^ were distributed throughout the cytoplasm in metaphase cells (Figure 6C; Movie S5), in contrast to a membrane-anchored MyrPalm-GFP control protein (Figure 6D; Movie S6). After anaphase onset, ECT2-WT accumulated at the spindle midzone, translocated to the plasma membrane and became concentrated at the equatorial periphery, as described previously (Su et al., 2011). ECT2-BRCT^TK^ accumulated at the cell periphery with timing similar to that of ECT2-WT. However, ECT2-BRCT^TK^’s localization to spindle midzone microtubule bundles (Figures 6C and 6E) and the protein’s enrichment at the equatorial plasma membrane (Figures 6C and 6F) were severely compromised. Image quantification revealed residual enrichment of ECT2-BRCT^TK^ at the equatorial membrane when compared to the profile of the membrane-anchored marker protein MyrPalm-GFP (Figure 6F). To quantify the equatorial accumulation of ECT2 during cytokinesis, we determined the protein intensity ratio at the equatorial membrane to the polar membrane over time (Figure S5A). Consistent with the earlier single time point analysis (Figure 6F), ECT2-BRCT^TK^ showed a strongly reduced but residual equatorial enrichment when compared to ECT2-WT over time (Figure S5A). Also, an accumulation of ECT2 around the midbody region following furrowing completion could be detected (Figure 6C; Movie S5). This concentration could be a consequence of the residual enrichment of the mutant protein at the congressing equatorial membranes. The MyrPalm-GFP control protein also showed equatorial enrichment at late stages of cytokinesis (Figure 6D; Figure S5A). This apparent equatorial accumulation was detected slightly earlier than it was for ECT2-BRCT^TK^. This is likely caused by a difference in the speed at which cells progress through cytokinesis (Figure S5B). Thus, it is possible that the congression of two membranes at the equator contributes to the detected late enrichment of ECT2 at this location.

Collectively, these data demonstrate that the mutations in the BRCT1 domain of ECT2 that disrupt binding to MgcRacGAP (Figure 5D) abrogate the recruitment of ECT2 to the spindle midzone and severely compromise the enrichment of the protein at the equatorial plasma membrane.

### ECT2-BRCT^TK^ Supports Cytokinesis in Human Cells

To examine the importance of ECT2’s targeting to the spindle midzone and equatorial membrane during cell division, we determined whether the ECT2-BRCT^TK^ transgene could support cytokinesis. Cell lines stably expressing AcFL-tagged and siRNA-resistant ECT2-BRCT^TK^ close to the level of the endogenous protein were compared with cell lines stably expressing different versions of ECT2 (Figure 7A; Figure S6A) (Su et al., 2011). Endogenous ECT2 was depleted by siRNA transfection (Figure 7A; Figure S6B), and the fraction of multi-nucleated cells was analyzed as an endpoint assay for prior cytokinesis failure. Cells expressing the AcFL tag only (AcFL), a GEF-defective ECT2 mutant (GEF^4A^), or a truncated ECT2 version lacking the PH domain and PBC (ΔPHΔTail) were converted into multi-nucleated cells following the depletion of endogenous ECT2 (Figure 7B). The cytokinetic defect induced by depletion of endogenous ECT2 could be fully rescued by expression of the wild-type ECT2 transgene. Unexpectedly, expression of the ECT2-BRCT^TK^ protein also potently rescued cytokinesis after depletion of endogenous ECT2 in two independent monoclonal cell lines (Figure 7B). Complete rescue was furthermore obtained using a pool of ECT2-BRCT^TK^ transgenic cells and two additional monoclonal cell lines (Figures S6C–S6E), suggesting that clonal effects do not account for the observed rescue.

We next examined the phenotype of BRCT mutant-expressing cells using live-cell imaging (Figures 7C and 7D). The quantification of cytokinetic phenotypes confirmed that ECT2-BRCT^TK^, in contrast to other defective ECT2 alleles, supports cytokinesis in the absence of endogenous ECT2, because most cells divided successfully (Figure 7D). The few ECT2-BRCT^TK^ cells that failed to divide still formed a cleavage furrow, which later regressed. In contrast, most cells lacking ECT2 or expressing defective ECT2 alleles failed to form a cleavage furrow. Thus, time-lapse studies indicate that the point mutations within ECT2’s BRCT1 domain that block binding to MgcRacGAP, abrogate midzone recruitment, and compromise enrichment of ECT2 at the equatorial membrane (Figures 5 and 6) do not prevent cleavage furrow formation and cytokinesis completion in most cells. Cell division in ECT2-BRCT^TK^ cells was still dependent on MgcRacGAP (Figure 7E), suggesting that the ECT2-BRCT^TK^ allele did not bypass the requirement for MgcRacGAP during cytokinesis.

We next determined whether the BRCT1 domain mutations and consecutive changes in ECT2 protein localization altered the profile of the contractile ring proteins RhoA and anillin along the periphery of anaphase cells. Depletion of endogenous ECT2 in the AcFL tag-only-expressing cells disrupted the cortical accumulation and equatorial enrichment of RhoA and anillin (Figure 7F). This phenotype was fully rescued in the ECT2-WT-expressing cells. The RhoA and anillin profiles in ECT2-BRCT^TK^ cells were undistinguishable from the profiles observed in ECT2-WT cells (Figure 7F).

In summary, our experiments demonstrate that ECT2-BRCT^TK^ supports the successful execution of cell division. This suggests that ECT2’s BRCT domain-mediated interaction with centralspindlin, ECT2’s bulk recruitment to the spindle midzone, and normal enrichment at the equatorial membrane are not essential for the correct assembly of the contractile ring and successful execution of cytokinesis in human somatic cells.

## Discussion

Optogenetic activation of RhoA at the plasma membrane can induce partial furrowing activity in a spindle- and cell-cycle-independent manner (Wagner and Glotzer, 2016). This finding highlights the importance of understanding the spatial and temporal control of RhoA activity during cytokinesis. The association of the RhoGEF ECT2 with the spindle midzone and equatorial plasma membrane have positioned the protein at the center of models aiming to explain how RhoA may be activated at the equator (Burkard et al., 2009, Fededa and Gerlich, 2012, Petronczki et al., 2007, Su et al., 2011, Wolfe et al., 2009, Yüce et al., 2005). Strong experimental data support the requirement of ECT2’s GEF activity during cytokinesis (Prokopenko et al., 1999, Rossman et al., 2005, Su et al., 2011). Here we manipulated the protein’s accumulation at the plasma membrane in space and time and disrupted the protein’s recruitment to the spindle midzone.

Using chemical genetic and optogenetic approaches, we show that the artificial targeting of ECT2 to the plasma membrane can replace the otherwise essential role of ECT2’s native membrane engagement domains and largely restore cytokinesis upon depletion of the endogenous protein. Thus, the key role of the PH domain and the polybasic cluster of ECT2 is to mediate the translocation of ECT2 to the plasma membrane. We conclude that the association of the RhoGEF ECT2 with the plasma membrane is an indispensable prerequisite for RhoA activation and cytokinesis in human cells and possibly most other animal cells.

Cell-cycle stage-specific photoactivation and TPA addition or washout experiments revealed that ECT2’s binding to the plasma membrane from the metaphase-to-anaphase transition onward is required and sufficient to support cell division. These experiments define the essential window of activity of ECT2 at the plasma membrane for cytokinesis.

ECT2 has been implicated in the establishment of a stiff mitotic cell cortex and timely mitotic cell rounding (Kunda and Baum, 2009, Matthews et al., 2012). Because ECT2’s engagement with the plasma membrane from the metaphase-to-anaphase onward suffices to drive cytokinesis, the formation of a stiff mitotic cell cortex during mitotic entry may not be essential for the execution of cytokinesis later. ECT2 thus activates RhoA for two temporally separate processes during cell division: mitotic cell rounding during prophase and cytokinetic furrow formation during anaphase.

The forced premature localization of ECT2 to the plasma membrane in metaphase increased the levels of downstream cortical cytokinetic regulators but did not result in premature ectopic furrow formation. Preventing the association of the RhoGEF ECT2 with the plasma membrane likely represents one of several parallel mechanisms by which Cdk1 prevents cleavage furrow formation before sister genomes have been partitioned. Forced membrane translocation of GEF-C1B, but not ECT2-C1B, in metaphase led to premature cortical contractility but was unable to restore cytokinesis in cells depleted of endogenous ECT2. These observations support the idea that the N-terminal part of ECT2, including the BRCT repeats, acts as an intramolecular inhibitor of RhoGEF function (Kim et al., 2005). Unrestrained uniform activation of RhoA around the cortex during cytokinesis could provide an explanation for the failure of GEF-C1B to restore cytokinesis upon TPA addition.

To test the spatial requirements of ECT2’s interaction with the plasma membrane, ECT2 was targeted to one area of the equatorial membrane in anaphase cells using optogenetics. Most cells in which the localized accumulation of ECT2 was observed developed a unilateral furrow at the point of illumination. This experiment suggests that the local presence of ECT2 at the equatorial plasma membrane is necessary to induce cleavage furrow formation. Because localized accumulation of ECT2 supported furrowing but not completion of cytokinesis, ECT2 and RhoA may have to be activated all around the equatorial cortex for successful cell division.

Illumination at the polar periphery was unable to induce ECT2 accumulation or furrowing activity at the cell pole. Instead, ECT2 accumulated at the cell equator, possibly via cytoplasmic diffusion of the activated protein or lateral diffusion of the CIBN-bound protein in the membrane, followed by preferential retention at the equatorial region. This was accompanied by equatorial furrowing in more than half of the cells. These observations suggest that ECT2’s membrane distribution could be subject to inhibition at the cell poles or to positive feedback control at the equator, two concepts worth exploring in the future. Artificial targeting of ECT2 to the plasma membrane resulted in furrow formation at the equator in anaphase but not at ectopic sites. This suggests that membrane engagement of ECT2, in contrast to RhoA activation (Wagner and Glotzer, 2016), is not sufficient to drive furrowing and that ECT2 might require an activator at the cell equator.

Mutations in the first BRCT repeat of ECT2 (BRCT^TK^) prevent binding of ECT2 to the centralspindlin subunit MgcRacGAP in cell extracts and ECT2’s accumulation at the spindle midzone. Although the mutant protein translocated to the cell periphery with similar timing as the wild-type counterpart, its enrichment at the equatorial membrane was also severely compromised. This demonstrates that ECT2’s recruitment to the spindle midzone depends on the BRCT domain-mediated interaction with centralspindlin. Furthermore, the centralspindlin-ECT2 interaction promotes the accumulation of RhoGEF at the equatorial plasma membrane. The association of ECT2 with centralspindlin could be a key element in the molecular mechanism the mitotic spindle uses to break cortical isotropy and define the cleavage plane in animal cells.

Despite ECT2-BRCT^TK^’s altered cellular distribution in anaphase cells, the mutated allele was able to fully support cleavage plane specification, contractile ring assembly, cleavage furrow ingression, and ultimately cytokinesis. These data indicate that the BRCT domain-mediated interaction of ECT2 with centralspindlin, as determined by co-immunoprecipitation; the recruitment of ECT2 to the midzone; and the protein’s normal enrichment at the equatorial plasma membrane are not essential for cytokinesis in otherwise unperturbed human cells.

The conclusions drawn earlier rely on data derived from an ECT2 allele carrying the mutations T153A and K195M in the phosphopeptide-binding groove formed by the protein’s BRCT repeats. Structural considerations and biophysical, cellular, and biochemical data (this work and (Wolfe et al., 2009, Zou et al., 2014) suggest that the mutations strongly inhibit the interaction of ECT2 with MgcRacGAP and the spindle midzone. Additional interactions between ECT2 and MgcRacGAP that are undetectable by co-immunoprecipitation might exist, such as the binding of the ECT2 BRCT repeats to non-phosphorylated MgcRacGAP (Yüce et al., 2005) or regulatory interactions between the C-terminal halves of both proteins (Zhang and Glotzer, 2015). We cannot exclude that these weak interactions or residual minor enrichment of ECT2 at the equatorial membrane and midzone contribute to the execution of cytokinesis in ECT2-BRCT^TK^ cells. However, given the strong defect in protein localization, we would expect at least a partial defect in contractile ring formation or cytokinesis in ECT2-BRCT^TK^ cells if midzone and equatorial membrane accumulation of ECT2 were directing the contractile RhoA zone or were essential for cytokinesis. Even such partial defects were not observed, despite the analysis of several independent clonal transgenic cell lines and transgenic cell pools. Furthermore, protein depletion data, as well as the use of defective ECT2 alleles, indicate that partial depletion of the endogenous ECT2 protein does not account for the absence of a phenotype in ECT2-BRCT^TK^ cells.

In the light of these observations, ECT2’s recruitment to midzone microtubules via MgcRacGAP and its normal enrichment at the equatorial membrane are unlikely to be the sole or primary signal for the formation and placement of the cleavage furrow in small somatic human cells. Previously proposed models are insufficient to explain how the spindle midzone stimulates equatorial furrow formation and will have to be revised (Fededa and Gerlich, 2012, Green et al., 2012, Mierzwa and Gerlich, 2014, Petronczki et al., 2007, Wolfe et al., 2009, Yüce et al., 2005). ECT2, PLK1, and MgcRacGAP are all essential for furrow formation (Bastos et al., 2012, Canman et al., 2008, Dechant and Glotzer, 2003, Jantsch-Plunger et al., 2000, Loria et al., 2012, Petronczki et al., 2007, Prokopenko et al., 1999, Tatsumoto et al., 2003, Yüce et al., 2005). However, our data suggest that the interpretation of the relevance of PLK1-mediated complex formation between ECT2 and MgcRacGAP might have been simplistic (Burkard et al., 2009, Petronczki et al., 2007, Wolfe et al., 2009). PLK1 likely has important substrates other than MgcRacGAP, whose phosphorylation contributes to the initiation of cytokinesis. Furthermore, our data on ECT2-BRCT^TK^ suggest that the non-phosphorylatable MgcRacGAP mutants employed previously either affect functions of MgcRacGAP other than binding to ECT2 or more potently inhibit the interaction with ECT2 compared with the BRCT^TK^ mutations.

The presence of ECT2 at the plasma membrane and its GEF activity are both essential for cell division. Our data suggest that the BRCT domain-dependent equatorial enrichment of ECT2 protein may not be crucial for cytokinesis. Thus, ECT2 could be activated exclusively at the cell equator to specify the cytokinetic furrow. Recent work has identified a role for centralspindlin in stimulating ECT2 GEF activity toward RhoA at the plasma membrane (Basant et al., 2015, Zhang and Glotzer, 2015). Consistent with a potential involvement of this mechanism, we found that cell division driven by ECT2-BRCT^TK^ requires the function of MgcRacGAP. In *C. elegans*, ECT2 is not concentrated at the equator like in human cells but distributed evenly across the cell envelope during anaphase (Chan and Nance, 2013, Motegi and Sugimoto, 2006). Defining a conserved mechanism that activates ECT2 specifically at the equatorial membrane to stimulate furrow formation could help unify models for cytokinesis in animal species.

While our work provides insights into the spatiotemporal regulation of cytokinesis, it also highlights the need to critically test models and to renew efforts to identify molecular mechanisms that can explain how the mitotic spindle apparatus specifies the cleavage plane and activates RhoA in animal cells. These mechanisms may act redundantly with ECT2’s equatorial enrichment and involve a second signal emerging from the midzone or astral microtubules (Bringmann and Hyman, 2005, Dechant and Glotzer, 2003, Werner et al., 2007, Zanin et al., 2013, Zhang and Glotzer, 2015). The generation of the ECT2-BRCT^TK^ model, in which the ECT2-midzone-recruitment dependent mechanism can be selectively inactivated, could facilitate the identification of redundant pathways in the future.

## Experimental Procedures

Additional information is provided in Supplemental Experimental Procedures.

### siRNA Transfection

Reverse siRNA transfections were performed using Lipofectamine RNAiMAX reagent (Invitrogen) according to the manufacturer’s protocol. The medium was changed 6 hr after transfection. The final concentration of siRNA in the medium was 20 nM. The following siRNA duplexes were used: control siRNA (non-targeting control [NTC]) (Thermo Scientific siGenome Non-Targeting siRNA #1 D-001210-01 and #4 D-001210-04), ECT2 siRNA (Thermo Scientific siGenome D-006450-02), and MgcRacGAP siRNA (Invitrogen Stealth HSS120934).

### Cell Synchronization and Drug Treatments

Phorbol ester treatment: 12-*O*-tetradecanoylphorbol-13-acetate (TPA, Sigma) at a final concentration of 10 nM, 250 nM, or 1 μM was added to cells for inducing the artificial membrane targeting of C1B-containing proteins. Equivalent volumes of the solvent DMSO were added to control samples. For the experiment shown in Figure 2B, cells were transfected with ECT2 siRNA and subsequently arrested in metaphase using the proteasome inhibitor MG132 (10 μM, Sigma) as described (Figure 2A) (Petronczki et al., 2007). Then, 45 min after MG132 washout and release into anaphase, either DMSO or 10 nM TPA was added to the medium and live-cell recording was initiated. The same protocol was followed for the experiment showed in Figure S2, but the concentration of TPA was changed to 250 nM. The TPA washout experiment shown in Figure 2E also followed the same protocol as described earlier for cells treated with DMSO and TPA. For cells treated with 10 nM TPA, along with MG132 TPA was washed away afterward, as shown in Figure 2D. To enrich the culture for metaphase cells for the experiment shown in Figure 3D and Figure S3B, cells were treated with 166 nM nocodazole (Sigma) for 4.5 hr. One hour after release from nocodazole, DMSO or 1 μM TPA was added. Cells were analyzed by immunofluorescence (IF) microscopy 5 min after DMSO or TPA addition. To enrich for anaphase cells for IF analysis (Figures 6B and 7F), 6 hr after siRNA transfection, the cells were treated for 24 hr with 2.5 mM thymidine. The cells were released from thymidine block for 9.5 hr and processed for IF analysis.

### Co-immunoprecipitation

HeLa Kyoto cells stably expressing AcFL, ECT2-WT, and ECT2-BRCT^TK^ were synchronously released to anaphase as described earlier and by Petronczki et al. (2007). Cells were collected 70 min after the release from MG132-induced metaphase block and lysed in buffer containing 20 mM HEPES, 150 mM NaCl, 0.1% Triton X-100, 5 mM MgCl_2_, 1 mM DTT, 1 mM PMSF, 1 μg/mL leupeptin, 1 μg/mL pepstatin, 1 μM microcystin, and 20 mM NaF. Lysates were then subjected to immunoprecipitation using GFP-Trap beads (Chromotek) and analyzed by immunoblotting.

### Statistical Methods

All graphs presented in this study were generated using GraphPad Prism v.6.0a. This software was also used for the statistical analysis. All datasets were collected as biological triplicates unless otherwise specified. Statistical tests used in each case are defined in the figure legends.

## Author Contributions

Conceptualization, K.K., K.-C.S., S.C.W., and M.P.; Methodology, K.K., K.-C.S., and M.P.; Formal Analysis, K.K.; Investigation, K.K.; Resources, K.K., K.-C.S., S.C.W., and M.P.; Writing – Original Draft, K.K.; Writing – Review & Editing, K.-C.S., S.C.W., and M.P.; Visualization, K.K.; Supervision, M.P.; Funding Acquisition, S.C.W. and M.P.

## Supplemental Information

Document S1. Supplemental Experimental Procedures and Figures S1–S6

Movie S1. Live-Cell Imaging of ECT2-C1B and ECT2-C1B^Q27G^ after TPA Treatment, Related to Figure 1C

Movie S2. Live-Cell Imaging of AcFL-C1B and AcFL-C1B^Q27G^ Proteins after TPA Treatment, Related to Figure 1C and Figures S1D and S1E

Movie S3. Live-Cell Imaging of Cry2-mCh-ECT2 with or without Blue-Light Illumination, Related to Figure 4C

Movie S4. Live-Cell Imaging of Cry2-mCh-ECT2 with Unilateral Blue-Light Illumination, Related to Figure 4E

Movie S5. Live-Cell Imaging of ECT2-WT and ECT2-BRCT^TK^ Localization during Cytokinesis, Related to Figure 6C

Movie S6. Live-Cell Imaging of MyrPalm-GFP Localization during Cytokinesis, Related to Figure 6D

Document S2. Article plus Supplemental Information
