# Supplementary material for: Plasma Membrane Association but Not Midzone Recruitment of RhoGEF ECT2 Is Essential for Cytokinesis
Source: Cell Rep. 2016 Dec 6;17(10):2672–86. doi: 10.1016/j.celrep.2016.11.029 (PMC5177604; doi:10.1016/j.celrep.2016.11.029)
Supplement: Document S1. Supplemental Experimental Procedures and Figures S1–S6 [file mmc1.pdf]

Cell Reports, Volume *17*

Supplemental Information

Plasma Membrane Association but Not Midzone Recruitment of RhoGEF ECT2 Is Essential for Cytokinesis

Kristýna Kotýnková, Kuan-Chung Su, Stephen C. West, and Mark Petronczki

Supplemental Figures

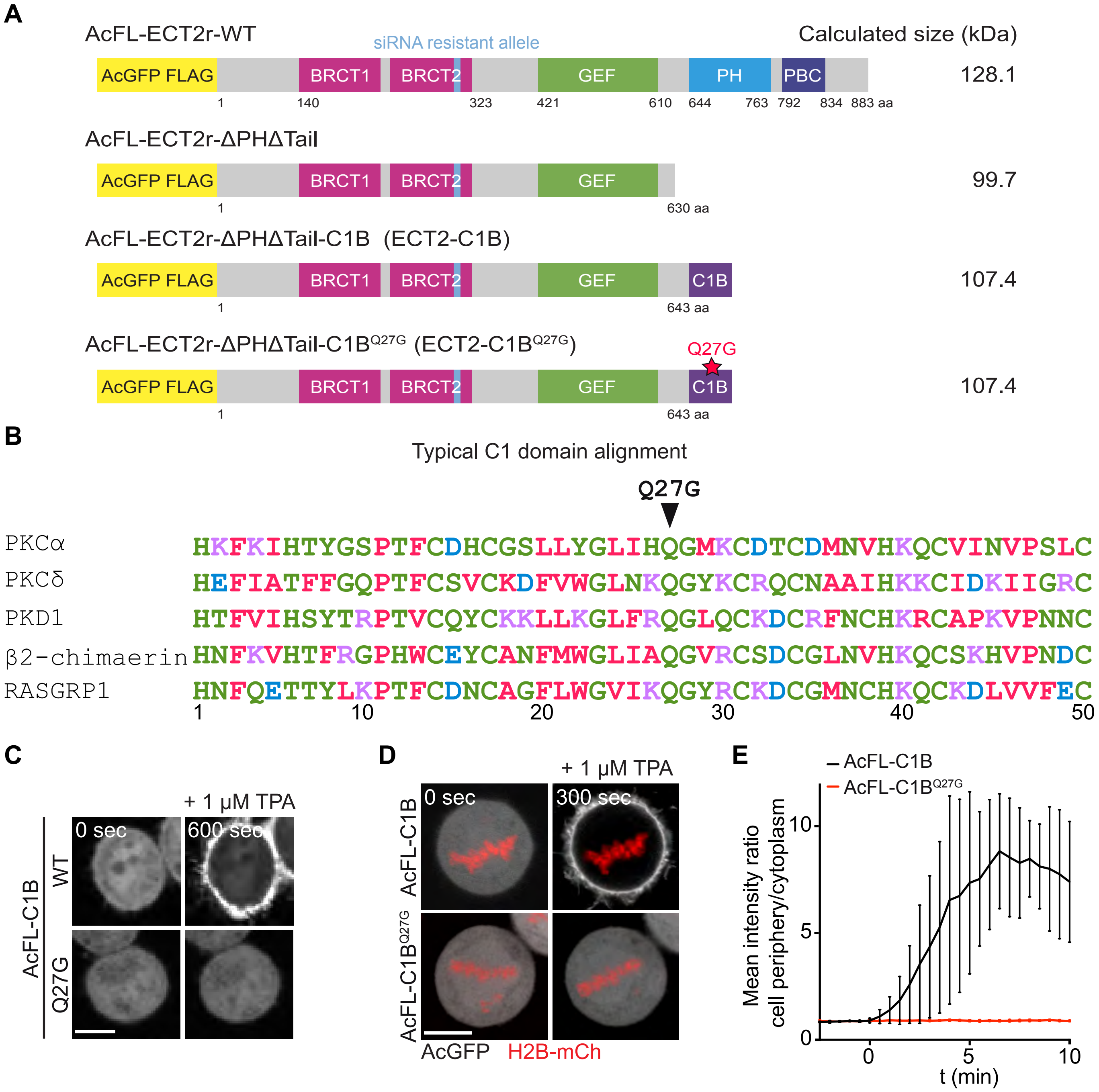

Figure S1. Artificial membrane targeting of ECT2 using C1B domain hybrid proteins (related to Figure 1)
(A) Schematic representation of the domain organization of different ECT2 constructs used to generate AcFL-tagged siRNA-resistant monoclonal cell lines for artificial ECT2 membrane targeting. Numbering of amino acid residues corresponds to their positions in human full-length ECT2 protein.
(B) Sequence alignment of human C1 domains from the indicated proteins. The first sequence belongs to the C1B domain from PKCα, which was used for construction of hybrid proteins. The residue glutamine 27, which was mutated to glycine to generate a version of C1B that no longer interacts with phorbol esters, is indicated.
(C) Stills from imaging of cells transiently transfected with GFP-tagged wild type or mutant C1B domains (AcFL-C1B). The cells were treated with 1 μM TPA 48 hours after transfection. t = 0 sec is set to the frame prior to TPA addition. The scale bars in this and the subsequent panels represent 10 μm.
(D) Frames from live-cell imaging of AcFL-C1B and AcFL-C1B^{Q27G} proteins. Cells were transiently transfected with plasmids encoding AcFL-C1B or AcFL-C1B^{Q27G} (white) and H2B-mCherry (red). Cells were treated with TPA and imaged 48 hours after transfection. t = 0 min is set to the time of TPA addition.
(E) Quantification of C1B domains translocation to the plasma membrane after the TPA treatment (as shown in D). Graph depicts peripheral to cytoplasmic ratio of the GFP signal. t = 0 secis set to the time of TPA addition. (n = 6 cells, lines represent mean ± SD).

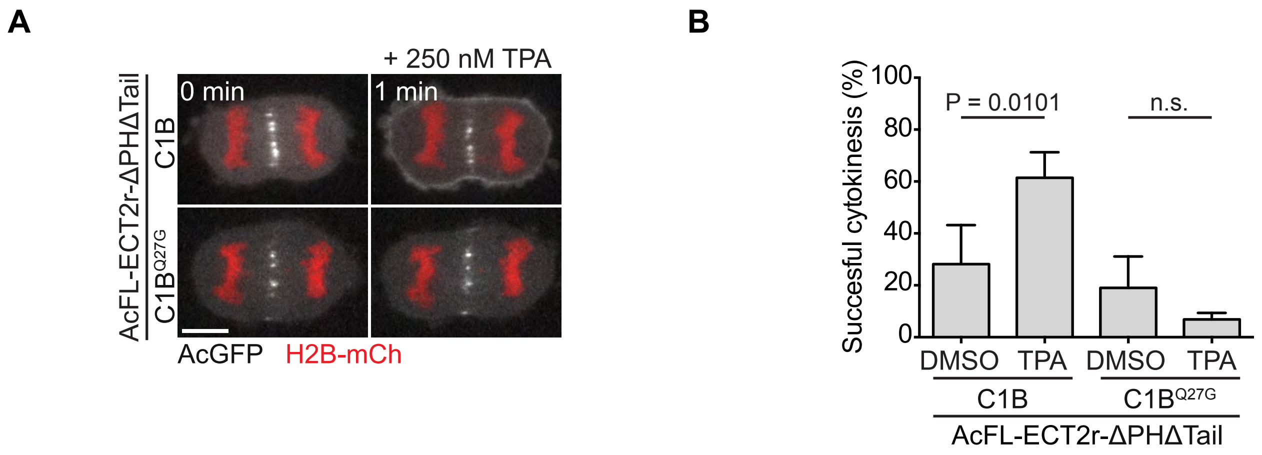

Figure S2. Plasma membrane association of ECT2 in anaphase suffices to support cytokinesis (related to Figure 2)
(A) Frames from live-cell imaging of the ECT2-C1B protein interacting with the plasma membrane after the TPA treatment. Stable cell lines expressing ECT2-C1B or ECT2-C1B^{Q27G} (white) were transiently transfected with H2B-mCherry (red). Cells were treated with DMSO or 250 nM TPA and imaged 48 hours after transfection. t = 0 min is set to the time of TPA addition. The scale bar represents 10 µm.
(B) Quantification of cytokinetic phenotypes for ECT2-C1B and ECT2-C1B^{Q27G} stable cell lines. Cells were transfected with ECT2 siRNA and synchronized in metaphase using the synchronization protocol depicted in Figure 2A. Cells were treated with DMSO or 250 nM TPA 45 minutes after release from the metaphase block and imaged using bright field microscopy. Mono-nucleated cells that were in metaphase at the beginning of recording were scored. ($n > 100$ each, bars represent mean ± SD of three independent experiments, Student's t test).

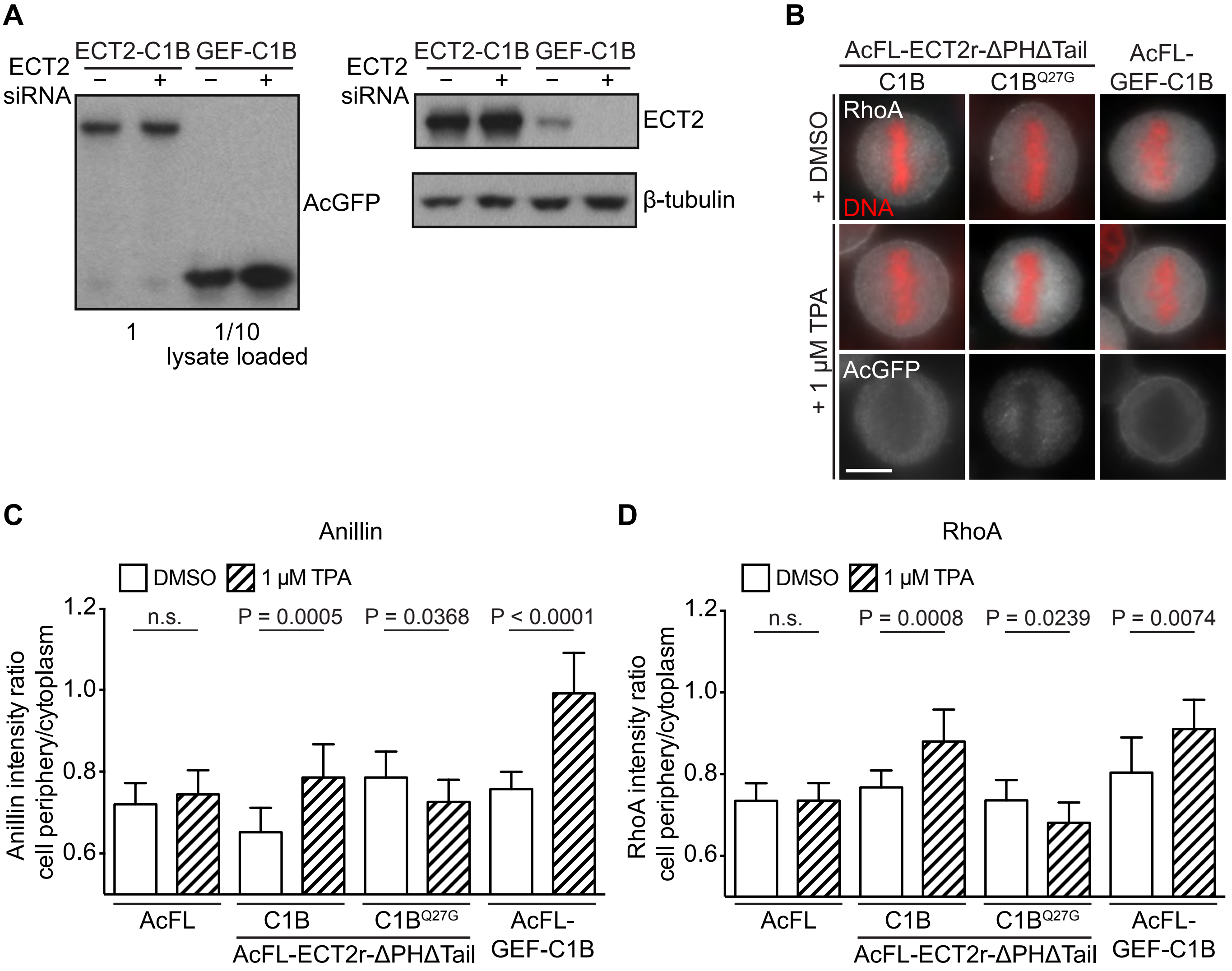

Figure S3. Forced membrane recruitment of ECT2 and its GEF domain in metaphase elicits signs of contractility (related to Figure 3)

(A) Immunoblot analyses of protein lysates from cells stably expressing ECT2-C1B and GEF-C1B proteins. Protein lysates were prepared 48 hours after transfection with NTC (-) or ECT2 siRNA (+). For the GEF-C1B sample 1/10 of the lysate was loaded for the blot on the left.

(B) IF analysis of cortical enrichment of RhoA in metaphase cells expressing the indicated transgenes. Cells were treated with nocodazole to enrich the population for prometaphase cells. 1 hour after nocodazole washout, the cells were treated with DMSO or TPA for 5 minutes, fixed and stained. The scale bar represents 10 µm.

(C) Quantification of cortical anillin enrichment in metaphase cells expressing indicated transgenes. Cells were treated with nocodazole to increase the fraction of prometaphase cells. 1 hour after nocodazole washout, the cells were treated with DMSO or TPA for 5 minutes, fixed and stained. Graph shows the peripheral to cytoplasmic anillin signal ratio (n = 10 cells, bars represent mean ± SD, Student's t test).

(D) Quantification of cortical RhoA enrichment in metaphase cells expressing indicated transgenes. Cells were treated and analysed as in (C). (n = 10 cells, bars represent mean ± SD, Student's t test).

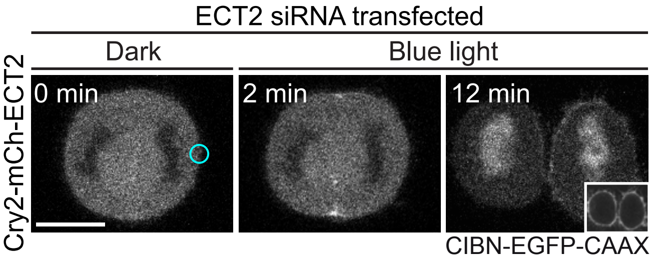

Figure S4. Optogenetic targeting of ECT2 to the polar part of plasma membrane does not induce ECT2 accumulation at the polar periphery (related to Figure 4)
Frames of live-cell imaging with unilateral blue-light illumination. Cells stably expressing CIBN-EGFP-CAAX (inset) were transfected with Cry2-mCh-ECT2 and ECT2 siRNA. Cells were imaged 24 hours after siRNA transfection and the plasma membrane translocation of Cry2-mCh-ECT2 was induced by unilateral illumination with a 488 nm laser within the circular region at the polar periphery as indicated. The scale bar represents 10 µm.

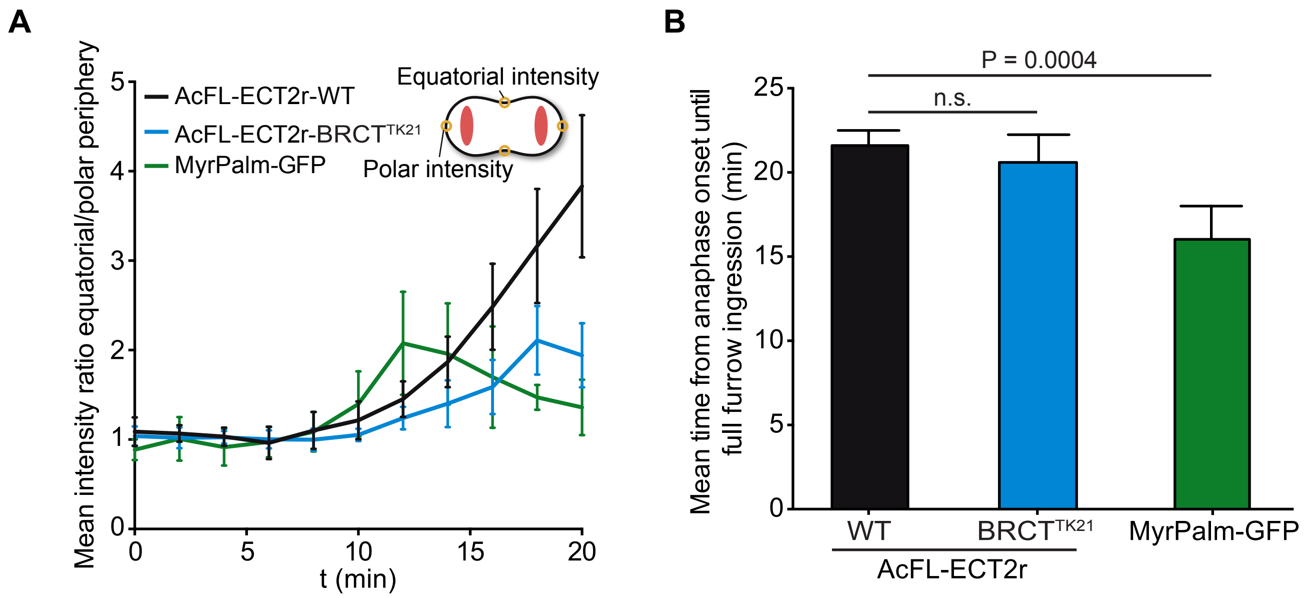

Figure S5. Mutations in BRCT1 domain of ECT2 disrupt the equatorial enrichment of the protein (related to Figure 6)
(A) Quantification of the equatorial enrichment of ECT2-WT, ECT2-BRCT^{TK} and MyrPalm-GFP during cytokinesis based on frames from confocal live-cell imaging. The graph shows the fluorescent intensity ratio between the equatorial and polar membrane (schematically depicted in the cartoon), measured from the metaphase-to-anaphase transition (t = 0 min) until complete furrow ingression. Data were obtained by measuring fluorescence intensity in small circular regions placed as shown on the cartoon on the right side. (n = 6 cells for ECT2-WT and MyrPalm-GFP and n = 10 cells for ECT2-BRCT^{TK}, lines represent mean ± SD).
(B) Analysis of the time from anaphase onset to completion of furrow ingression from cells analysed in (A). (n = 6 cells for ECT2-WT and MyrPalm-GFP and n = 10 cells for ECT2-BRCT^{TK}, bars represent mean ± SD, Student's t test).

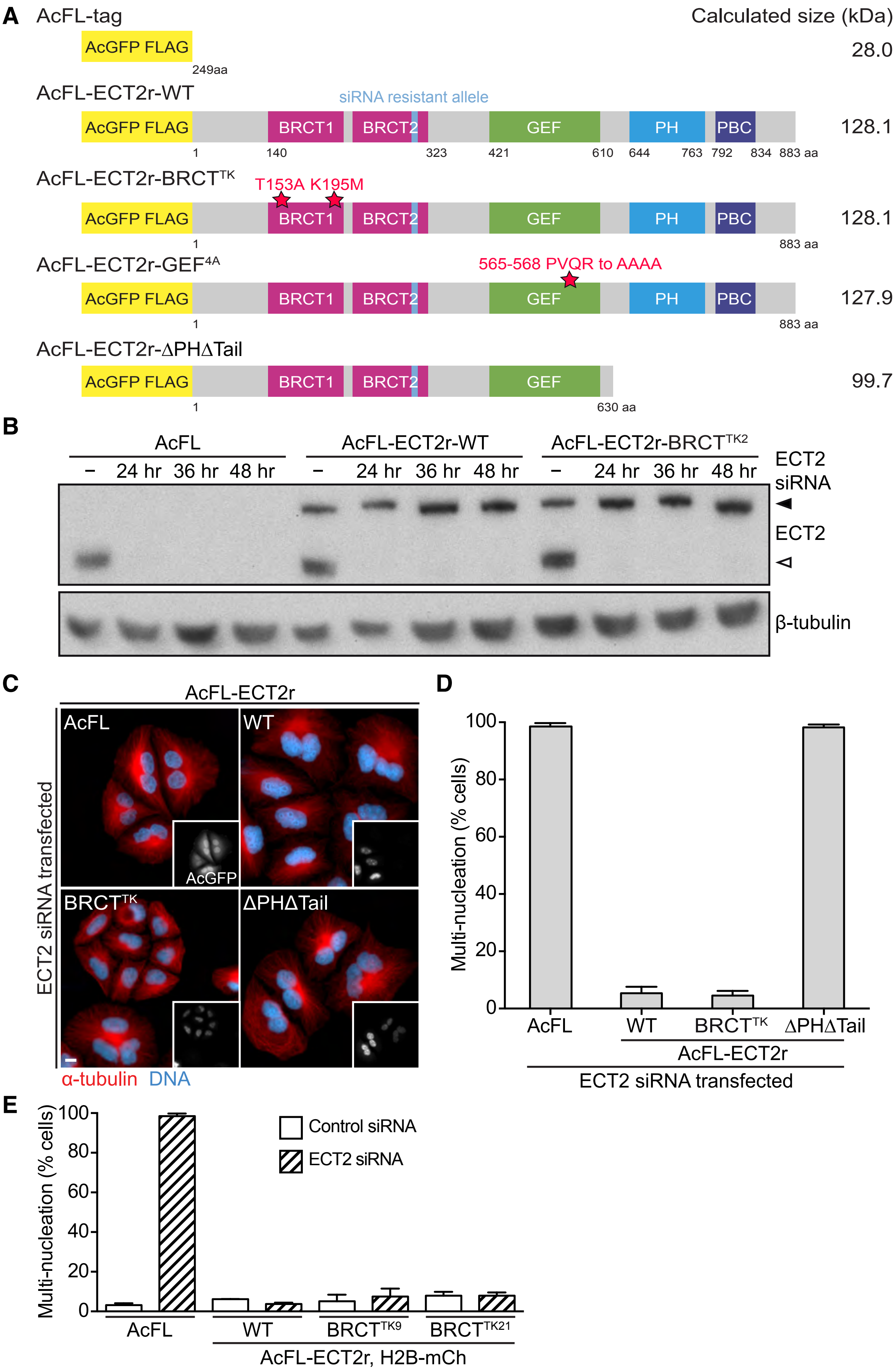

Figure S6. Spindle midzone recruitment of ECT2 is not essential for cytokinesis in human cells (related to Figure 7)

(A) Schematic representation of the domain organization of different AcFL-tagged siRNA-resistant ECT2 constructs used to generate stably expressing monoclonal cell lines for studying the cytokinetic role of ECT2-BRCT^{TK}. Numbering of amino acid residues corresponds to their positions in human full-length ECT2 protein.
(B) Immunoblot analysis of protein lysates from monoclonal cell lines stably expressing the indicated transgenes showing efficiency of endogenous ECT2 depletion by siRNA. Protein lysates were prepared 48 hours after transfection with NTC (-) or 24, 36 and 48 hours after transfection with ECT2 siRNA. Endogenous ECT2 protein and transgenic AcFL-ECT2r are indicated by open and filled arrowheads respectively.
(C) IF analysis of pool cell lines stably expressing indicated transgenes. Cells were transfected with ECT2 siRNA and 48 hours after transfection analysed by IF. The scale bar represents 10 μm.
(D) Quantification of cytokinetic phenotypes from panel C. Multi-nucleation levels were analysed by IF 48 hours after siRNA transfection. Only cells expressing transgenic ECT2 were scored. (n > 200 each, bars represent mean ± SD of three independent experiments).
(E) Quantification of cytokinetic phenotype of cells stably co-expressing H2B-mCh with indicated transgenes.
Multi-nucleation levels were analysed by IF 48 hours after siRNA transfection. (n > 300 cells each, bars represent mean ± SD of three independent experiments).

Supplemental Movie Legends

Movie S1. Live-cell imaging of ECT2-C1B and ECT2-C1B^{Q27G} after TPA treatment (related to Figure 1C)

HeLa Kyoto cell lines stably expressing ECT2-C1B or ECT2-C1B^{Q27G} (green) were transiently transfected with H2B-mCherry (red). Cells were treated with 1 µM TPA and imaged 48 hours after transfection. Frames were acquired every minute. t = 0 min is set to the time of TPA addition.

Movie S2. Live-cell imaging of AcFL-C1B and AcFL-C1B^{Q27G} proteins after TPA treatment (related to Figure 1C, S1D and S1E)

HeLa Kyoto cells were transiently transfected with plasmids encoding AcFL-C1B or AcFL-C1B^{Q27G} (green) and H2B-mCherry (red). Cells were treated with 1 µM TPA and imaged 48 hours after transfection. Frames were acquired every 30 seconds. t = 0 sec is set to the frame prior to TPA addition.

Movie S3. Live-cell imaging of Cry2-mCh-ECT2 with or without blue-light illumination (related to Figure 4C)

HeLa Kyoto cells stably expressing CIBN-EGFP-CAAX were transfected with Cry2-mCh-ECT2 (red) and ECT2 siRNA and imaged 24 hours after siRNA transfection. Activation was performed by illumination with a 488 nm laser within two small circular regions at the equatorial periphery as marked in the Figure 4C. Frames were acquired every minute. t = 0 min is set to the time of blue-light illumination.

Movie S4. Live-cell imaging of Cry2-mCh-ECT2 with unilateral blue-light illumination (related to Figure 4E)

HeLa Kyoto cells stably expressing CIBN-EGFP-CAAX were transfected with Cry2-mCh-ECT2 and ECT2 siRNA and imaged 24 hours after siRNA transfection. Activation was performed by illumination with a 488 nm laser within one small circular regions at the equatorial periphery as marked in the Figure 4E. Frames were acquired every minute. t = 0 min is set to the time of blue-light illumination.

Movie S5. Live-cell imaging of ECT2-WT and ECT2-BRCT^{TK} localization during cytokinesis (related to Figure 6C)

HeLa Kyoto cell lines stably co-expressing ECT2-WT or ECT2-BRCT^{TK} (green) with H2B-mCherry (red) were transfected with ECT2 siRNA and imaged 48 hours after transfection. Frames were acquired every 2 minutes. Time point t = 0 min was set to the metaphase-to-anaphase transition.

Movie S6. Live-cell imaging of MyrPalm-GFP localization during cytokinesis (related to Figure 6D)

HeLa Kyoto cell lines stably expressing MyrPalm-GFP (green) transfected with H2B-mCherry (red) were imaged 48 hours after transfection. Frames were acquired every 2 minutes. Time point t = 0 min was set to the metaphase-to-anaphase transition.

Supplemental Experimental Procedures

Plasmids for expression in human cells

To generate plasmids encoding transgenes for expression in human cells, the transgenes were inserted into pIRESpuro3 vector (Clontech), containing an N-terminal AcGFP-FLAG tag (GFP from *Aequorea coerulescens* coupled to a single FLAG epitope) using AgeI and EcoRI restriction sites. Resistance to

ECT2 siRNA was achieved by introduction of synonymous nucleotide changes into the ECT2 (Su et al., 2011). Point mutations, deletions and hybrid transgenes were created using site-directed mutagenesis and PCR. To create the hybrid ECT2-C1B plasmid, residues 644 to 883 of ECT2 were replaced by the C1B domain of human PKCa (amino acids 102 to 151) (kindly provided by P. Parker). pH2B_mCherry_IRES_neo3 (Addgene Plasmid 21044) was kindly provided by Daniel Gerlich (Steigemann et al., 2009). pCry2PHR-mCh-N1(Addgene Plasmid 26866) and pCIBN(deltaNLS)-pmGFP (Addgene Plasmid 26867) were obtained from Addgene (Kennedy et al., 2010). To generate the Cry2-mCh-ECT2 plasmid, residues 1 to 643 of ECT2 were N-terminally fused to the photolyase homology region of cryptochrome 2 protein (Cry2PHR, residues 1 to 498) coupled to a mCherry tag (Kennedy et al., 2010).

DNA transfection and generation of monoclonal stable cell lines

HeLa Kyoto cells were grown as described (Petronczki et al., 2007). Plasmids were transfected into HeLa Kyoto cells using FuGENE 6 transfection reagent (Promega). To select and maintain cell lines stably expressing pIRESpuro3 plasmids, the medium was supplemented with 0.3 µg/ml puromycin (Sigma). To select for the expression of pIRESneo3 plasmids (mCherry tagged transgenes) or pCIBN(deltaNLS)-pmGFP, 400 µg/ml geneticin (G418, Gibco) was added to the cell medium. Monoclonal cell lines were isolated after two weeks of antibiotic selection. Cell lines were characterized by IF microscopy and western blotting. Cell lines expressing AcFL, AcFL-ECT2r, AcFL-ECT2r-ΔPHΔTail, AcFL-ECT2r-GEF^{4A} and AcFL-ECT2r together with H2B-mCherry are described elsewhere (Su et al., 2011). For transient expression, the X-tremeGENE 9 DNA transfection reagent (Roche) was used.

Immunofluorescence microscopy (IF)

Cells were fixed in ice cold methanol (-20ºC) for 2 hours to overnight (Figure 1D, 3C, 3D, 6B, 7B, 7F and S6C) in 4% PFA (paraformaldehyde, Thermo) diluted in PBS for 10 minutes at 37ºC (AcFL-tag sample from Figure 7B and S6C) or with 10% TCA (trichloroacetic acid) on ice for 15 minutes (Figure 7F (RhoA samples) and S3B) before being processed for immunofluorescence microscopy as described (Lenart et al., 2007). Images were acquired on a Zeiss Axio Imager M1 or M2 microscope using a Plan Neofluor 40x/1.3 oil objective lens (Figure 1D, 3C, 7B and S6C) or Plan Apochromat 63x/1.4 oil objective lens (Figure 3D, 6B, 7F and S3B) (both from Zeiss) equipped with an ORCA-ER camera (Hamamatsu) and controlled by Volocity 6.1 software (Perkin Elmer).

Antibodies and dyes

The following primary antibodies were used: mouse monoclonal anti-AcGFP (Clontech JL8, WB 1:1000), rabbit polyclonal anti-ECT2 (raised against ECT2 1-421 aa, WB raw serum 1:2000, IF 1:5000) (Su et al., 2011), rabbit monoclonal anti-β-tubulin (Cell Signaling 9F3, WB 1:2000), goat polyclonal anti-MgcRacGAP (Abcam ab2270, WB 1:500) rabbit polyclonal anti-AcGFP (Clontech 632592, IF 1:2000), rat monoclonal α-tubulin (AbD Serotec MCA78G, IF 1:1000), mouse monoclonal anti-MKLP1 (Santa Cruz Biotechnology 24, IF 1:500), rabbit polyclonal anti-anillin (kindly provided by Michael Glotzer, IF 1:2000) (Piekny and Glotzer, 2008) and mouse monoclonal anti-RhoA (Santa Cruz Biotechnology 26C4, IF 1:75). Secondary antibodies conjugated to Alexa Fluor 488 or Alexa Fluor 594 (Molecular Probes, IF 1:500) were used for immunofluorescence detection. DNA was stained with 4',6-diamidin-2-fenylindol (DAPI) at 1 µg/ml (Molecular Probes). HRP-conjugated secondary antibodies (polyclonal goat anti-mouse P0447 and polyclonal anti-rabbit P0488, Dako) were used at 1:5000 dilution to detect protein signals on PVDF membrane.

Live-cell imaging

Before imaging, the cell medium was changed to sterile-filtered imaging medium (CO_2-independent medium [Gibco], 20% FCS, 1% Pen Strep and 0.2 mM L-glutamine [Gibco]). Images for Figure 1C, 3B, 6C, 6D and S2A were obtained at 37ºC on a PerkinElmer ERS Spinning disc system equipped with a Nikon TE2000 microscope, an Apo TIRF 60x/1.49 oil objective lens (Nikon), a CSU22 spinning disc scanner (Yokogawa) and a IEEE1394 Digital CCD C4742-80-12AG camera (Hamamatsu), controlled by Volocity 5.5.1 software (Perkin Elmer). Images for Figure 4B, 4C, 4E and S4 were acquired at 37ºC

on Invert780 Zeiss LSM multi-photon confocal system equipped with Zeiss Axio Observer.Z1 microscope, a Plan-Apochromat 63x/1.46 oil objective lens and a GaAsP spectral detector all controlled by Zen2012 software. The plasma membrane interaction of Cry2-mCh-ECT2 was triggered by scanning with a 488 nm laser (Figure 4B). More spatially selective targeting of Cry2-mCh-ECT2 was triggered by illumination with a 488 nm laser inside two small circular regions of 20 pixels in diameter placed at opposite locations of the equatorial cortex every 5 minutes (Figure 4C). Unilateral targeting of Cry2-mCh-ECT2 was achieved by illumination inside one small circle every 2 minutes at one location of the equatorial cortex (Figure 4E) or at the polar periphery (Figure S4). Images for Figure S1C were acquired at 37ºC on an Olympus FV1000D (Inverted Microscope IX81) laser confocal scanning microscope using a PlanApoN 60x/1.40 oil Sc objective lens (Olympus) controlled by FV10-ASW software. Phase contrast images in Figure 1E, 2B and 7C were obtained by using an IncuCyte FLR integrated live-cell imaging system (Essen Bioscience). Cells were imaged every 10 minutes in regular cell growth medium.

Image quantification

Images were quantified using ImageJ software version 1.46r (http://rsbweb.nih.gov/ij/). The cell periphery signal for Figure 6E was obtained by measuring the intensity profile of the AcGFP signal along a line manually placed along the cell periphery in ImageJ (function Plot profile). The profiles were made relative to a maximum value for each set of measurements. The mean peripheral intensity for RhoA and anillin in Figure 7F was obtained the same way. The cytoplasmic signal was measured by averaging the signal in three manually selected circular regions with a diameter of 50 pixels. The mean background signal was obtained by averaging the signal of three manually selected circular regions with a diameter of 50 pixels outside of the cell and the value was subtracted from the cell periphery and cytoplasmic values. After that, the ratio of cell periphery to cytoplasmic average signals was calculated for each cell analyzed. Mean intensity ratios of cell periphery to cytoplasm were measured for Figure S1E, S3C and S3D. The membrane signal values were obtained by averaging six circular regions of 9 pixels in diameter at the cell periphery. The cytoplasmic and background signals were obtained and the ratio was calculated as described above. For Figure S5B, the ratio of mean AcGFP signal at the equatorial periphery to the polar periphery was determined as described above. The equatorial periphery signal was obtained by averaging two circular regions of 12 pixels in diameter at the cell periphery at both sides of the furrow. The polar periphery signal was obtained by averaging two circular regions of 12 pixels in diameter at the cell periphery at both cell poles. Mean intensity ratios of midzone to cytoplasm were determined for Figure 6E as described above with the exception that midzone values that were obtained by measuring signal intensity in a rectangular equatorial region excluding the membrane with a constant width of 10 pixels. Images were processed with ImageJ 1.46r and Adobe Photoshop CS5.1. Structural alignment in Figure 5A was done using MatchMaker tool in the UCSF Chimera software version 1.8.1.

Supplemental References

Kennedy, M.J., Hughes, R.M., Peteya, L.A., Schwartz, J.W., Ehlers, M.D., and Tucker, C.L. (2010). Rapid blue-light-mediated induction of protein interactions in living cells. Nat. Methods *7*, 973-975.

Lenart, P., Petronczki, M., Steegmaier, M., Di Fiore, B., Lipp, J.J., Hoffmann, M., Rettig, W.J., Kraut, N., and Peters, J.M. (2007). The small-molecule inhibitor BI 2536 reveals novel insights into mitotic roles of polo-like kinase 1. Curr. Biol. *17*, 304-315.

Petronczki, M., Glotzer, M., Kraut, N., and Peters, J.M. (2007). Polo-like kinase 1 triggers the initiation of cytokinesis in human cells by promoting recruitment of the RhoGEF Ect2 to the central spindle. Dev. Cell *12*, 713-725.

Piekny, A.J., and Glotzer, M. (2008). Anillin is a scaffold protein that links RhoA, actin, and myosin during cytokinesis. Curr. Biol. *18*, 30-36.

Steigemann, P., Wurzenberger, C., Schmitz, M.H., Held, M., Guizetti, J., Maar, S., and Gerlich, D.W. (2009). Aurora B-mediated abscission checkpoint protects against tetraploidization. Cell *136*, 473-484.

Su, K.C., Takaki, T., and Petronczki, M. (2011). Targeting of the RhoGEF Ect2 to the equatorial membrane controls cleavage furrow formation during cytokinesis. Dev. Cell *21*, 1104-1115.
