# Supplementary figures and images for: Plasma Membrane Association but Not Midzone Recruitment of RhoGEF ECT2 Is Essential for Cytokinesis
Source: Cell Rep. 2016 Dec 6;17(10):2672–86. doi: 10.1016/j.celrep.2016.11.029 (PMC5177604; doi:10.1016/j.celrep.2016.11.029)

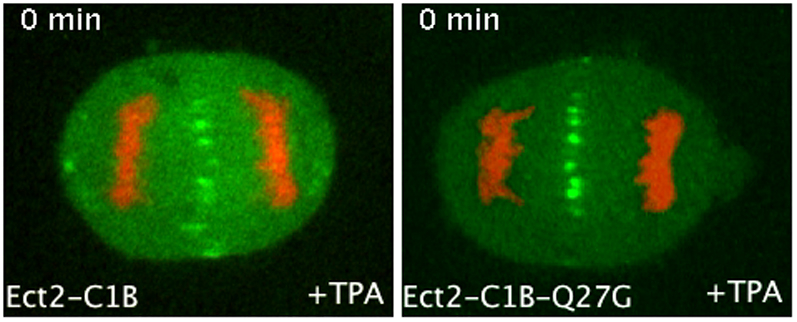

Supplement: Movie S1. Live-Cell Imaging of ECT2-C1B and ECT2-C1BQ27G after TPA Treatment, Related to Figure 1C [file mmc2.jpg]

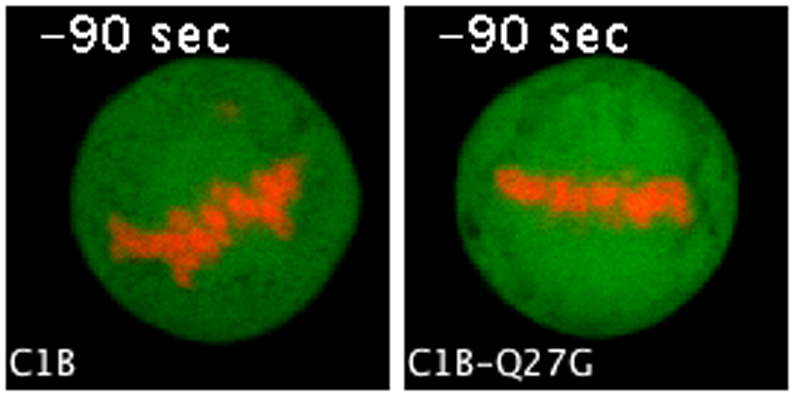

Supplement: Movie S2. Live-Cell Imaging of AcFL-C1B and AcFL-C1BQ27G Proteins after TPA Treatment, Related to Figure 1C and Figures S1D and S1E [file mmc3.jpg]

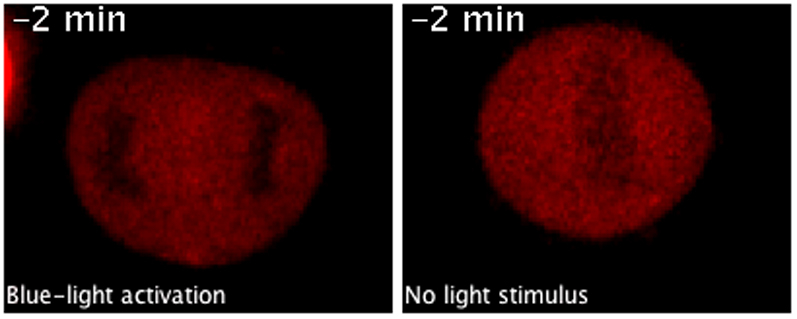

Supplement: Movie S3. Live-Cell Imaging of Cry2-mCh-ECT2 with or without Blue-Light Illumination, Related to Figure 4C [file mmc4.jpg]

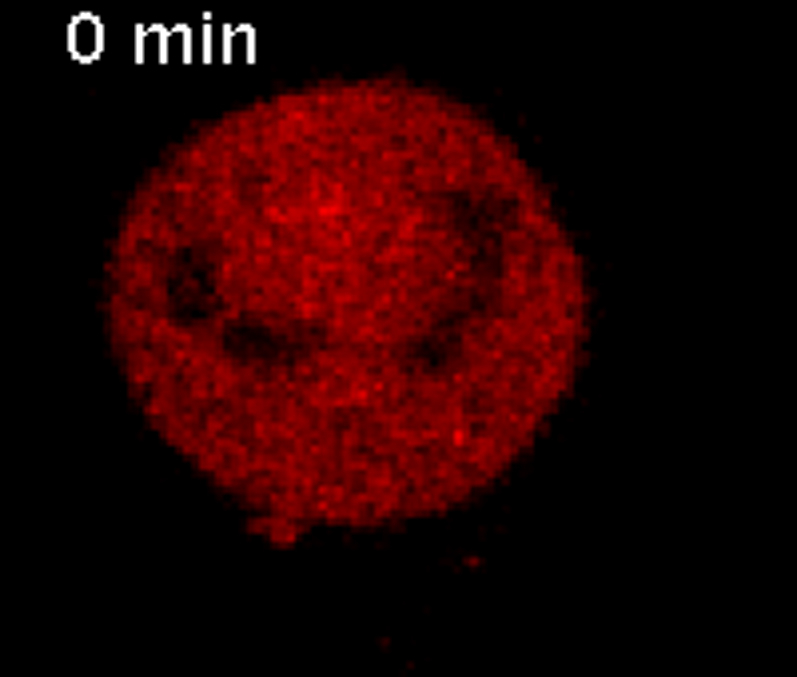

Supplement: Movie S4. Live-Cell Imaging of Cry2-mCh-ECT2 with Unilateral Blue-Light Illumination, Related to Figure 4E [file mmc5.jpg]

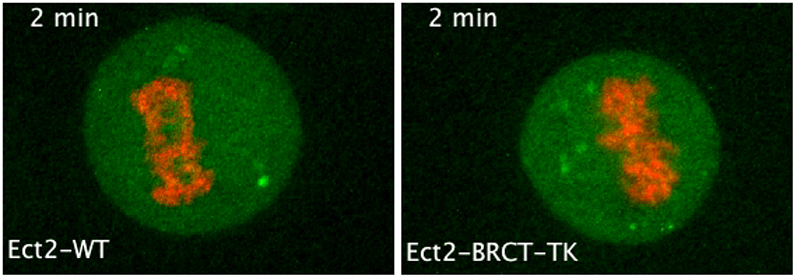

Supplement: Movie S5. Live-Cell Imaging of ECT2-WT and ECT2-BRCTTK Localization during Cytokinesis, Related to Figure 6C [file mmc6.jpg]

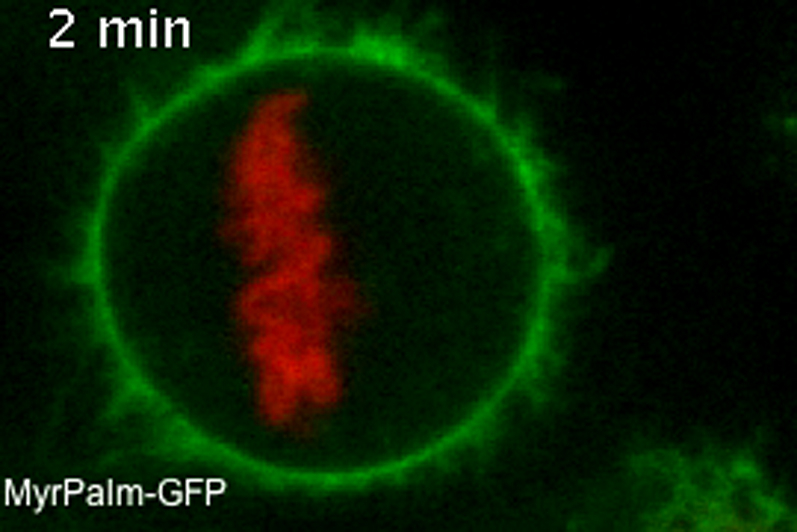

Supplement: Movie S6. Live-Cell Imaging of MyrPalm-GFP Localization during Cytokinesis, Related to Figure 6D [file mmc7.jpg]
